# Computational validation and network pharmacology reveal the cardioprotective and hypolipidemic potential of *Arisaema Jacquemontii Blume* via molecular docking, metadynamics, DFT, and MM/PBSA analyses

**DOI:** 10.3389/fbinf.2026.1856040

**Published:** 2026-06-12

**Authors:** Manisha Shah, Sivakumar Arumugam

**Affiliations:** Department of Bio-Sciences, School of Bio Sciences and Technology, Vellore Institute of Technology, Vellore, Tamil Nadu, India

**Keywords:** *Arisaema Jacquemontii Blume*, atherosclerosis, cardiovascular disease, DFT, molecular docking simulation, network pharmacology, TNF-alpha

## Abstract

**Introduction:**

Despite the recognized therapeutic value of plant-derived compounds, *Arisaema jacquemontii Blume*, a traditional Himalayan herb, remains largely unexplored for cardiovascular applications. Based on ethnomedicinal evidence and pharmacological studies demonstrating antioxidant and protein kinase inhibitory activities, which are closely associated with cardiovascular protection, we hypothesized that *A. Jacquemontii Blume* may exert cardioprotective effects through the modulation of oxidative stress, inflammatory signaling, and lipid metabolism pathways relevant to atherosclerosis.

**Methods:**

An integrated computational framework combining network pharmacology, tissue-specific gene expression profiling, transcriptomic analysis, and Bayesian Network via Splitting Average was employed to identify potential therapeutic targets. Molecular docking, 300 ns molecular dynamics simulations, MM/PBSA binding free energy calculations, density functional theory (DFT) analysis, metabolic prediction (XenoSite and SMARTCyp), and *in silico* bioactivity and ADMET assessments were performed to evaluate the pharmacological potential of the identified phytochemicals.

**Results:**

TNF-alpha and ESR1 were identified as key putative therapeutic targets. Molecular docking revealed favorable binding affinities of Gamma-sitosterol and Phenol 2,5-bis(1,1-dimethylethyl)- toward these proteins compared with co-crystallized reference ligands. Molecular dynamics simulations and MM/PBSA analyses indicated stable protein–ligand interactions throughout the 300 ns simulations. DFT analysis provided insights into the electronic properties and charge-transfer characteristics of the phytochemicals. Metabolic prediction suggested a low risk of reactive intermediate formation and favorable CYP3A4 stability, while bioactivity and ADMET profiling indicated potential roles in regulating cholesterol homeostasis, oxidative stress, and vascular inflammation.

**Conclusion:**

Although the findings are based exclusively on computational analyses and require experimental validation, they provide a robust predictive framework supporting the potential hypolipidemic and cardioprotective relevance of *A. Jacquemontii Blume*. These results establish a strong rationale for further experimental investigation of its therapeutic potential in atherosclerosis.

## 1 Introduction

Cardiovascular disease (CVD) is a broad term encompassing conditions impacting the heart and blood vessels and represents one of the leading global causes of mortality. CVD is primarily driven by atherosclerosis, the buildup of fatty substances in the arteries (Jebari-Benslaiman et al., 2022). In addition to its global prevalence, gender-related factors also influence the clinical manifestation and outcomes of CVD, often in opposite ways (Regitz-Zagrosek and Gebhard, 2023). By 2030, CVD is expected to account for more than 23 million deaths globally (Lozano et al., 2012), highlighting the pressing necessity for effective strategies in both prevention and treatment (Tefera et al., 2017).

Atherosclerosis is a long-term inflammatory disorder marked by the intimal collection of immune cells, lipids and fibrous material within the artery walls, serving as a major driver of cardiovascular disease and leading to severe complications such as heart attack and strokes (Ross, 1999). The pathogenesis of atherosclerosis is driven by the multifaceted interaction of oxidative stress, disruptions in lipid metabolism, and immune-related inflammatory processes, all of which contribute to plaque buildup and impaired vascular function (Tabas et al., 2015). Despite advances in conventional therapies, the multi-factorial nature of atherosclerosis demands innovative strategies that can address multiple pathways simultaneously (Libby, 2002).

Even with standard treatment options in place, natural compounds are attracting growing interest as potential therapeutics due to their high bioavailability, minimal adverse effects, and the capability to influence specific mechanisms within the body. Many, plant-derived compounds often provide a promising starting point for developing treatment (Stan et al., 2021). Across many cultures, medicinal plants have been traditionally used for centuries to treat a wide range of illnesses. Some of the most well-known drugs, such as aspirin from *Filipendula ulmaria* and quinine from *Cinchona pubescens,* were derived from plants (Elisha et al., 2017). Similarly, numerous plants, including *Terminalia arjuna, Ocimum sanctum* (holy basil), and *Allium sativum* (garlic), have demonstrated cardioprotective effects, highlighting the importance of natural sources in CVD management. However, because the chemical scaffolds and molecular mechanisms of these well-characterized plants have been extensively mapped, there is a pressing need to explore untapped ethnomedicinal resources to identify additional bioactive compounds with potential therapeutic relevance.

Among these, *Arisaema Jacquemontii Blume*, a plant native to Afghanistan, Kashmir, Pakistan, East Asia, and the Himalayas (Baba and Malik, 2015), has demonstrated multiple bioactivities, including anti-inflammatory, antioxidant, anti-proliferative, protein kinase inhibitory, and anti-cancer properties (Jeelani et al., 2010; Kaur et al., 2006; Ovais et al., 2018; Tabassum et al., 2019; Zahra et al., 2017). Despite these reported pharmacological properties, its role in cardiovascular disease remains largely underexplored. The rationale for selecting *A. Jacquemontii Blume* lies in its combination of bioactivities particularly antioxidant, anti-inflammatory, and protein kinase-modulating effects which are mechanistically relevant to the pathophysiology of atherosclerosis. These properties are closely associated with key processes such as oxidative stress, vascular inflammation, and dysregulated signaling pathways involved in plaque progression. Given this mechanistic alignment, *A. Jacquemontii Blume* represents a promising yet underexplored candidate for investigation in cardiovascular disease. Based on these established bioactivities, it is reasonable to hypothesize that *A. Jacquemontii Blume* may exert cardioprotective effects through modulation of oxidative stress, inflammatory signaling, and lipid metabolism pathways, thereby providing a biochemical rationale for its investigation as a potential source of bioactive compounds for atherosclerosis.

**FIGURE 1:**
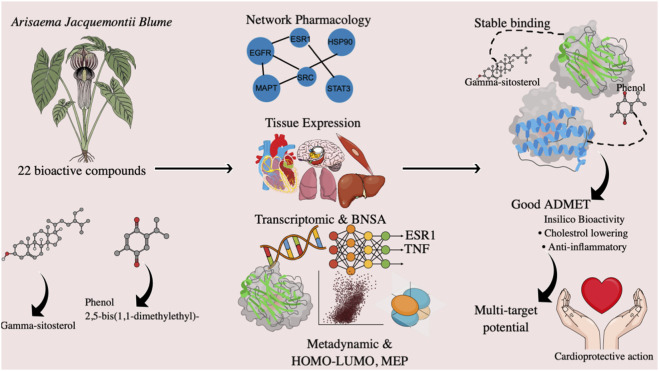
A graphical representation of the research methodology, outlining the sequential steps undertaken from data acquisition to result interpretation.

In the present study, we aimed to systematically explore the cardioprotective potential of bioactive compounds from *A. Jacquemontii Blume* using an integrative *in silico* framework. By combining network pharmacology, tissue-specific expression profiling, transcriptomic analysis, and molecular modeling, we sought to identify key molecular targets and elucidate the predictive mechanisms underlying their anti-atherosclerotic effects. In contrast to traditional target-based studies, the key regulatory hubs in this work were identified through a data-driven approach using Bayesian Network via Splitting Average (BNSA) of human transcriptomic data. This unbiased methodology prioritized Tumor Necrosis Factor-alpha (TNF-alpha) and Estrogen Receptor 1 (ESR1) as the central mechanistic pillars governing vascular inflammation and lipid homeostasis, respectively. By computationally evaluating these targets, this approach provides a robust, hypothesis-generating foundation for the rational development of plant-derived multi-target therapeutics and advances our understanding of natural compounds in combating complex cardiovascular diseases. Figure 1 provides a flowchart summarizing the complete study workflow.

## 2 Computational methodology

### 2.1 Screening of active compounds of *Arisaema jacquemontii Blume*

A comprehensive literature review identified 22 active compounds extracted from various parts of *A. Jacquemontii Blume*, including leaves (1 compound), pulp (3 compounds), seed (3 compounds), stem (4 compounds), and rhizome (11 compounds) (Shehzadi et al., 2022). Canonical SMILES strings for these 22 active phytochemicals were acquired from the PubChem chemical database (https://pubchem.ncbi.nlm.nih.gov). These structures were subsequently analyzed via SwissTargetPrediction (http://www.swisstargetprediction.ch/) (Daina et al., 2019) using a ligand-based reverse screening approach to identify potential human targets. To ensure the reliability of predictions based on the 2D Tanimoto index and 3D Electroshape 5D principles, a strict structural filtering protocol was implemented. Specifically, Triallylmethylsilane was excluded due to a heavy atom count below the required minimum (<5), while Propanenitrile, 3-(methylthio)- and Octane, 1-(propylthio)- were omitted as the absence of similar active compounds in the ChEMBL database precluded accurate similarity-based mapping. Furthermore, Phenol, 2,5-bis (1,1-dimethylethyl-) was treated as a single entry to eliminate redundancy arising from its presence in multiple plant extracts. This systematic filtering resulted in 18 unique bioactive compounds, whose predicted protein targets were subsequently harmonized to *Homo sapiens* gene symbols using the UniProt database to ensure consistent nomenclature for the construction of the plant-disease interactome.

### 2.2 Identification of disease-related gene

Information on human genes linked to cardiovascular disease was retrieved from the “OMIM (https://www.omim.org/)” and “GeneCards (https://www.genecards.org/),” databases. To ensure a targeted and relevant gene pool, the search was conducted using the keyword “heart disease”, and limiting species to “*Homo sapiens*”. After merging the gene lists from both databases and removing any duplicate genes, we obtained 16577 unique targets. To understand the overlap between the predicted targets of *A. Jacquemontii Blume* and these disease-specific targets, we created a Venn diagram. This analysis revealed 601 shared genes implicated in biological processes relevant to the disease pathology.

### 2.3 Generation of the protein interaction map

To elucidate the shared gene targets between *A. Jacquemontii Blume* and disease-related gene targets, a Venn diagram analysis was employed, revealing 601 common genes. These shared genes were mapped using the STRING database platform (https://string-db.org/; (Szklarczyk et al., 2021); with the parameters set to “*Homo sapiens*” for organism and a strict filtering threshold where the minima required interaction score was set to the “highest confidence” level (0.900). Cytoscape (version 3.10.1) facilitated the visualization and analysis of the generated protein interaction map (protein–protein interaction network). For PPI network construction and hub gene identification, two Cytoscape plugins- CytoNCA and CytoHubba were employed. The CytoNCA was employed to evaluate the key nodes in the network using five centrality measurements, including, subgraph centrality (SC), betweenness centrality (BC), closeness centrality (CC), degree centrality (DC), and eigenvector centrality (EC). A stringent selection criterion was established whereby genes exhibiting SC, DC, EC, BC, and CC values strictly exceeding the median across all five metrics were prioritized for further visual analysis.

The filtered network was further analyzed using CytoHubba, which applies multiple topological algorithms to identify key regulatory nodes. The top-ranked genes from each algorithm were compared, and overlapping candidates were selected to identify the most robust hub genes within the network. Using this integrative approach, six key targets were identified. This stepwise network pharmacology pipeline, incorporating high-confidence interaction thresholds, multi-parameter topological filtering, and algorithmic consensus, ensures systematic prioritization of biologically relevant targets.

### 2.4 Generation of a compound target-organs location network

Since the *in vivo* metabolism of *A. Jacquemontii Blume* remains unclear, investigating its potential benefits for cardiovascular disease requires analyzing tissue and organ-specific mRNA expression profiles. To address this, gene expression data were sourced from the Gene Atlas dataset within the BioGPS database (http://biogps.org), which provides microarray-based mRNA expression data across 84 organs and tissues (Prevoršek et al., 2010). For each compound target, mRNA expression was measured in 84 organs and tissues, followed by the calculation of average expression for individual tissues and for all tissues combined. Tissues exhibiting mRNA expression levels above the overall average were selected for further analysis (Suh and An, 2017). Finally, target-organ interaction networks were constructed using Cytoscape to visualize the tissue-specific expression patterns of the compound targets. To integrate tissue-specific expression data with network pharmacology results, the hub genes identified from the PPI network were mapped onto their corresponding tissue expression profiles. This enabled the assessment of whether key network-derived targets exhibit biologically relevant expression patterns in cardiovascular and associated tissues, thereby supporting their functional relevance in disease-specific mechanisms.

### 2.5 GO and KEGG enrichment inference

Enrichment analyses were performed using two independent platforms, g:Profiler (https://biit.cs.ut.ee/gprofiler/gost;, and Metascape (https://metascape.org/gp/index.html#/main/step1;, to characterize the functional roles of the identified hub genes. These tools employ distinct computational algorithms, enabling cross-verification of results and enhancing confidence in the findings. For this study, *Homo sapiens* was used as the reference organism. The g:Profiler platform offers broad compatibility with various species, gene identifiers, and evidence sources. Gene Ontology (GO) annotations were used to classify genes based on cellular components (CC), molecular functions (MF) and biological processes (BP). Pathway-based enrichment analysis was carried out using curated resources, including KEGG, Reactome, and WikiPathways, and additional protein-related information was obtained from the Human Protein Atlas and CORUM databases. Gene annotations was performed via g:Profiler, applying the g:SCS (Set Counts and Sizes) algorithm for multiple testing correction to control false positive, with a significance threshold set at 0.05. To further elucidate gene functions, we conducted additional functional enrichment analysis using Metascape (Kolberg et al., 2023; Zhou et al., 2019).

## 3 Gene expression profile data collection

In this study, gene expression data related to cardiovascular disease (GSE236251) (Kucherenko et al., 2023) were accessed from the Gene Expression Omnibus (GEO) repository provided by NCBI (https://www.ncbi.nlm.nih.gov/geo/). We used “heart disease,” and “*Homo sapiens* (organism)” as the keyword in the GEO NCBI database. With these search parameters, 2048 results were found. The expression profiling by high-throughput sequencing was selected according to the following rules: the sample must contain tissue from a healthy heart and a diseased heart; the patients did not receive any treatment; and the sample size should be not less than 10 samples. Under these conditions, we identified one dataset suitable for further analysis. Although another dataset met some of these criteria, it was excluded due to discrepancies in expression data and limited accessibility, as a substantial portion of the required data was not publicly available and required author-specific access. The use of a rigorously filtered dataset ensures that the identified differentially expressed genes are more likely to reflect underlying disease pathology rather than treatment-related effects, thereby supporting data quality and biological relevance. For downstream analysis, raw data for 29 specimens were obtained from GSE236251, including 8 healthy heart donors (control), 5 samples with left heart disease without pulmonary hypertension, and 15 samples with pulmonary hypertension due to left heart disease (diseased heart).

### 3.1 Screening of differentially expressed genes (DEGs)

We performed differential gene expression analysis using the “DESeq2” (version 1.36.0) (Love et al., 2014) in “Bioconductor”, which is based on the negative binomial distribution. To compare gene expression, data from healthy hearts and hearts with pulmonary hypertension caused by left heart disease were analyzed. Genes exhibiting a log2 fold change ≥0.5 and an adjusted p-value <0.05 were classified as significant DEGs. The distribution of DEGs were visualized with a volcano plot generated in R Studio using the ggplot library.

### 3.2 Gene set enrichment analysis

We conducted Gene Set Enrichment Analysis using GSEA software (version 4.3.2) (http://www.broadinstitute.org/gsea/index.jsp) (Subramanian et al., 2005) to analyze the gene expression dataset GSE236251, which consists of 28 samples. Only normalized counts were included in the data matrix for analysis. To fully characterize the biological pathways linked to our gene set, Gene Set Enrichment Analysis (GSEA) was conducted, using Human Ensembl Gene IDs as the reference platform “(ftp.broadinstitute.org://pub/gsea/annotations_versioned/Human_Ensembl_Gene_ID_MSigDB.v2022.1.Hs.chip;)”. Our analysis focused on identifying enriched processes in both healthy and diseased hearts by analyzing two key databases: KEGG pathways “(c2.cp.kegg.v2022.1.Hs.symbols.gmt)” and Hallmark modules “(h.all.v2022.1.Hs.symbols.gmt)”.

### 3.3 *De novo* transcription factor

Motif discovery was performed using the “Simple, Thorough, Rapid, Enriched Motif Elicitation” (STREME) and “Multiple Em for Motif Elicitation” (MEME), transcriptional factor (TF) motif analysis was conducted on DEGs (Bailey, 2021; Bailey and Elkan, 1994). Two types of enrichment analyses were performed: independent and relative. For the analysis, the gene sequences were truncated to include regions around the transcription start site (TSS) to ensure that the key regulatory regions were captured. Specifically, for each gene, a 2000 bp region upstream and downstream of the TSS was extracted, resulting in a total sequence length of 4,000 bp. If the TSS was located at the gene start or end, the sequences were extended by 4,000 bp in the opposite direction to maintain uniformity across all sequences. This method of truncation ensured that both upstream and downstream regulatory elements were preserved while focusing on the core region around the TSS. These truncated sequences were then analyzed for TF binding motifs using STREME and MEME (Pravallika and Rajasekaran, 2024).

*De novo* motifs (E-value ≤1.0, p-value ≤0.05) were screened against the HOCOMOCO Human v11 CORE (human DNA), *Homo sapiens* (DNA-encoded), and the Eukaryote DNA (vertebrates) databases using Tomtom. Matches with FDR q-values <0.25 and p-values ≤0.05 were deemed significant and used for further analysis.

### 3.4 Gene regulatory network assessment

The gene regulatory network (GRN) was constructed with the “Bayesian Network Splitting Average (BNSA)” method (Liu et al., 2009). This strategy is specifically designed to handle high-dimensional transcriptomic data by mitigating overfitting. In terms of implementation, the BNSA approach works by randomly splitting the dataset into multiple subsets, independently learning a Bayesian network structure for each subset, and then averaging these sub-networks to form a highly robust consensus network. The differentially expressed gene (DEG) data matrix was discretized into binary values, with 0 denoting median expression and 1 denoting expression above the median. The networks and the corresponding adjacency matrices were produced using the “Hillclimbsearch” algorithm and the Bayesian Information Criterion (BIC) score. GRNs were visualized and recreated in Cytoscape using adjacency matrices containing of nodes (genes) and edges (interactions) derived from the BNSA results. To rigorously validate the computational interactions predicted by the BNSA model, a dual-correlation validation protocol was implemented. The co-expression scatter plots were produced via utilizing ggpubr (version 0.5.0) and ggplot2 (version 3.4.0) packages based on z-scores derived from the normalized counts of patient samples. A p-value cut-off of 0.05 was used to further filter the DEGs using the Pearson and Spearman correlation scores. The edges in the GRNs were adjusted based on the p-value threshold, with varied thicknesses to represent the significance of correlations (Pravallika and Rajasekaran, 2024):

- “Lower thickness: indicates significant p-value for either the Spearman or Pearson correlation coefficient”.
- “Medium thickness: indicates significant p-value for both correlations but with a correlation coefficient less than 0.7”.
- “Highest thickness: indicates significant p-value for both correlations with correlation coefficients greater than 0.89”.

Lastly, the GRNs were analyzed with Cytoscape (v3.10.1) to characterize key network features.

### 3.5 Molecular docking procedure verification

#### 3.5.1 Protein and ligand preparations for molecular docking

A computational strategy was employed to examine the binding interactions of 18 prioritized phytochemicals from *A. Jacquemontii Blume* against two regulatory targets identified via GRN analysis. For clarity, Phenol, 2,5-bis(1,1-dimethylethyl-) is referred to as the phenol-derived compound throughout the manuscript. The 3D structures of these bioactives were retrieved from PubChem in SDF format and energy-minimized using the MMFF94 force field via Open Babel to ensure optimal low-energy conformations (Halgren, 1999; O’Boyle et al., 2011).

For the biophysical validation, high-resolution crystal structures of Tumor Necrosis Factor-alpha (TNF-alpha; PDB ID: 2AZ5, 2.10 Å) and Estrogen Receptor 1 (ESR1; PDB ID: 7UJW, 2.00 Å) were retrieved from the RCSB Protein Data Bank (Berman, 2000). Both proteins were expressed in an *Escherichia coli* system. The native co-crystallized ligands ID 307 for TNF-alpha (He et al., 2005), representing a small-molecule TNF-alpha binding inhibitor structurally related to assembly disruptors such as SPD-304, and ID R3V for ESR1 (Hosfield et al., 2022), a lasofoxifene derivative, were extracted during preparation and utilized as internal reference standards (hereafter referred to as TNF-alpha-standard and ESR1-standard). These co-crystallized ligands were employed to establish a benchmarking framework for docking analysis. Re-docking of the native ligands into their respective binding sites was performed to validate the docking protocol and ensure accurate reproduction of experimental binding poses. Subsequently, the binding affinities and interaction profiles of the phytochemicals were interpreted relative to these reference ligands, enabling a comparative assessment of their binding potential. Protein pre-processing was executed using AutoDockTools (v4.2.6) (Morris et al., 2009). The structural refinement workflow included the removal of solvent molecules and heteroatoms, inclusion of polar hydrogen atoms, the refinement and annexing of missing atoms, and the assignment of Kollman charges. The finalized structures were exported in PDBQT format for high-throughput screening. Virtual screening was executed using AutoDock Vina (v1.1.2) (Trott and Olson, 2010), utilizing a specialized scoring function to evaluate the binding affinities of the bioactive constituents against the prepared targets.

#### 3.5.2 Grid box

To identify the optimal docking area, a spherical selection was centered on the binding site of the co-crystallized inhibitors using BIOVIA Discovery Studio, enabling the precise determination of the active site coordinates. The search space for AutoDock Vina was defined using a 40 × 40 × 40 Å grid box centered on the native ligand positions. For TNF-alpha (PDB ID: 2AZ5), the docking site was centered at coordinates x = −19.4096, y = 74.6507, and z = 33.8495 (Shah and Arumugam, 2024), while for ESR1 (PDB ID: 7UJW), the center was set at x = 1.8053, y = 48.0678, and z = 61.9807. The docking procedure was automated using a custom Perl script to ensure consistency across the bioactive repertoire. To support the protocol, the native co-crystallized ligands (ID 307 and ID R3V) were removed and re-docked into their respective protein cavities to confirm the algorithm’s ability to replicate experimental binding poses. The resulting complexes were visualized and analyzed using BIOVIA Discovery Studio 2021 and PyMOL to evaluate key intermolecular interactions (Dassault Syst mes BIOVIA, 2017).

### 3.6 Molecular dynamic (MD) simulation

Molecular dynamics simulations is a robust computational approach for substantiation of inferences derived from the molecular docking studies, it provides a detailed insight into the complexes intricate atomic conformational details regarding the ligand’s compatibility with the protein of interest when simulated for a expanded span of time.The MD simulations was carried out using GROMACs platform for both the complexes, with input files preparation performed via the CHARMM-GUI Solution Builder (Lee et al., 2019). The simulation framework was developed by solvating each complex in a TIP3P water box with buffer of 15 Å in all spatial dimensions. To mimic local niche ionic conditions and attain charge neutrality, 0.15 M sodium chloride (NaCl) was solvated into the solvation mixture, and the overall system charge was homogenized. The relaxation phase proceeded with two concurrent stages of energy minimization followed by a single equilibration phase. During equilibration, the system’s temperature was maintained at 310 K via Nose–Hoover thermostat, with synchronized pressure development of 1.0 bar using semi-isotropic pressure coupling. The LINCS algorithm was used to apply bond constraints, and non-bonded interaction were handled through the Verlet cutoff method. post this, the production run was initiated for 300 nanoseconds using a 2-femtosecond time step, accumulating approximately 150 million integration steps with each nanosecond translating to 10 frames. Ligand topology and parameters were generated via Ligand Reader & Modeler module found integrated in CHARMM-GUI server, which employs the CHARMM General Force Field (CGenFF) to create force field-compatible input files (Kim et al., 2017). Simulations were performed using GROMACS version 2022.4 (Bauer et al., 2022), and trajectories were analyzed to assess structural stability through RMSD, RMSF, Rg, and hydrogen bonding. To ensure statistical validation, standard deviations for all structural parameters were rigorously calculated across the 300-ns trajectory frames. Furthermore, the explicit specification of environmental variables, the TIP3P water model, CGenFF, and thermodynamic algorithms ensures the complete reproducibility of this simulation protocol.

### 3.7 Metadynamic analysis

To further compliment the inferences derived from the prior assessments, a combined analysis employing “Principal Component Analysis (PCA) and Free Energy Landscape (FEL) mapping”. The Geo_measure 0.9 plugin in PyMOL was employed to ensure precise structural interpretation (Kagami et al., 2020). FELs were produced using Root Mean Square Deviation (RMSD) as abscissa and Radius of Gyration (Rg) values as ordinate, computed across each protein–ligand complex during the molecular dynamic simulations. RMSD served to quantify structural deviations relative to the reference conformation, whereas Rg provided information about the compactness and spatial distribution of the molecular system. These FEL plots highlighted thermodynamically stable conformational states as well as transitional intermediates, offering a comprehensive thermodynamic perspective on complex stability and conformational dynamics. Additionally, “Dynamic Cross-Correlation Matrix (DCCM)” analysis was exercised to examine patterns of correlated and anti-correlated motions between Cα atoms of the protein in response to ligand binding. This analysis elucidated inter-residue communication pathways and domain movements, providing important intricacy into the structural plasticity and dynamic interaction mechanisms of protein–ligand complexes (TNF-alpha–Gamma-Sitosterol and ESR1-alpha-Phenol, 2,5-bis (1,1-dimethylthyl-) and their respective standard (native ligand) (Yousaf et al., 2023).

### 3.8 MM-PBSA analysis for binding free energy (ΔG) calculation

Among computational methods, the Molecular Mechanics Poisson–Boltzmann Surface Area (MM/PBSA) is commonly employed to determined protein–ligand binding free energy. To achieve this, the gmx_MMPBSA tool (https://valdes-tresanco-ms.github.io/gmx_MMPBSA/v1.6.0/) was utilized to perform energy evaluations. This method computes several energy components, including electrostatic interactions (ΔE_ele_), van der Waals forces (ΔE_vdW_), and solvation energies, which are divided into polar (ΔGGB) and non-polar (ΔGSA) contributions (Valdés-Tresanco et al., 2021). To ensure the statistical validity and reproducibility of the binding predictions, the MM/PBSA energy calculations were averaged across multiple sampled frames from the stable molecular dynamics trajectories, ensuring that the final thermodynamic values robustly account for dynamic conformational fluctuations rather than a single static state. The total binding free energy (ΔG_bind_) is determined by combining these terms as follows

$$\Delta G_{\text{bind}}=\Delta G_{\text{solvation}}\;\left(\Delta \text{GSA}+\Delta \text{GGB}\right)+\Delta G_{\text{potential}}\;\left(\Delta E_{\text{ele}}+\Delta E_{\text{vdw}}\right)$$

The solvation energy (ΔG_solv_) accounts for polar and non-polar effects, where the non-polar component is estimated using the solvent-accessible surface area (SASA).

The total binding free energy was computed according to the following equation:

$$\Delta G_{\text{bind}}=\Delta G_{\text{complex}}\;–\;\left(\Delta G_{\text{protein}}+\Delta G_{\text{ligand}}\right)$$

In this context, ΔGcomplex denotes the overall binding free energy of the protein–ligand system, while ΔGprotein and ΔGligand refer to the individual free energies of the unbound protein and ligand, respectively. To further dissect the molecular interactions, a energy decomposition analysis was performed. This enabled the identification of specific amino acids residues within the binding pocket that significantly contribute for the stabilization and affinity of the complexes formed with Gamma-sitosterol and Phenol 2,5-bis(1,1-dimethylethyl)- (a phenol-derived compound). Understanding these residue-level contributions provides valuable insight into the anti-inflammatory and anti-atherosclerotic potential of the phytochemicals. Residues exhibiting high energetic contributions are likely essential for facilitating binding interactions and stabilizing the ligand within the active site.

## 4 Quantum chemical analysis

Density Function Theory (DFT) serves as a robust computational approach for probing the electronic properties of biologically relevant molecules. In this study, DFT was employed to explore the electronic characteristics of the two phytochemicals, Gamma-sitosterol and phenol-derived compound. The initial step involved geometry optimization of these compounds, performed with Gaussian 09W employing the B3LYP/6-31G(d) level of theory, ensuring energetically stable molecular conformations. To identify potential reactive regions, molecular electrostatic potential (MEP) maps were generated. Furthermore, key electronic descriptors, including frontier molecular orbitals (FMOs), namely the highest occupied molecular orbital (HOMO) and lowest unoccupied molecular orbital (LUMO) were examined. These were complemented by the calculation of global reactivity parameters to assess the chemical behavior and stability of the selected molecules.

## 5 Metabolic stability and CYP450-mediated biotransformation

Evaluating metabolism studies is essential for assessing the effectiveness and safety of drug candidates (Mukherjee et al., 2015). Cytochrome P450 (CYP) enzymes are key player in the biotransformation of various compounds, with variations in their activity have been implicated with heightened risk of cardiovascular disease (Elbekai and El-Kadi, 2006). To assess metabolic stability and biotransformation sites, we utilized XenoSite (https://xenosite.org/). This free web-based tool predicts sites of metabolism (SOM) and reactivity (SOR) based on neural network models trained on over 680 *in vivo* compounds. XenoSite offers quick processing and can identify reactive sites that interact with DNA and proteins (Matlock et al., 2015). Additionally, we employed SmartCYP (https://smartcyp.sund.ku.dk/about) (Rydberg et al., 2010) another free application, to identify metabolic hotspots by analyzing carbon oxidation states and predicting reactive sites using density functional theory (Rydberg et al., 2013). We also assessed the SASA of individual atoms to estimate metabolic susceptibility with the 2DSASA algorithm, refining our predictions of potential metabolic sites.

## 6 *In silico* pharmacokinetics, bioactivity, and toxicity evaluation

The potential biological activities of chemical compounds can be inferred from their interactions with biological systems, covering a wide range of effects. In silico approaches provide an efficient alternative to traditional laboratory based *in vitro* and *in vivo* methods for predicting bioactivity. One such tool, PASS (Prediction of Activity Spectra for Substances) (PASS Online) (https://www.way2drug.com/passonline/) is a web-based platform that applies structure–activity relationship (SAR) modeling to estimate the probability of biological activities of compounds. This tool aids in evaluating the therapeutic potential of drug-like compounds by analyzing two key parameters: Probability of Activity (Pa) and Probability of Inactivity (Pi). These probabilities, spanning from 0 to 1, are derived from training datasets of known biologically active compounds (Filimonov et al., 2014). A higher Pa indicates a stronger likelihood of biological activity, while a higher Pi suggests inactivity. In the PASS Online scoring system, the biological activity of a molecule is predicted on a scale from 0 (inactive) to 1 (active). Additionally, pkCSM (https://biosig.lab.uq.edu.au/pkcsm/) (Pires et al., 2015) is utilized to forecast ADMET (Absorption, Distribution, Metabolism, Excretion, and Toxicity) properties leveraging graph-based signatures and predictive modeling strategies. This computational approach aids in assessing the pharmacokinetic profile of compounds, facilitating informed drug development decisions. For toxicological evaluation, the OSIRIS Property Explorer (https://www.organic-chemistry.org/) was utilized. OSIRIS predicts drug-relevant properties and categorizes potential risks using a color-coded system. Toxicity risks, such as mutagenicity, tumorigenicity, irritation, or reproductive toxicity, are highlighted in red, indicating high-risk concerns. Conversely, properties that align with drug-like behavior are marked in green, signifying favorable characteristics (Sander, 2001).

## 7 Results and discussion

### 7.1 Network pharmacology-based analysis

#### 7.1.1 Active ingredient and disease-related gene identification from *Arisaema jacquemontii Blume*

Through a comprehensive literature review, we identified 22 bioactive compounds reported from *A. Jacquemontii Blume* (Shehzadi et al., 2022)*,* as detailed in Supplementary Table 1. After applying the structural filtering and redundancy criteria described in the Materials and Methods (Section 2.1), 18 unique bioactive constituents were prioritized for target prediction. Reverse screening of these compounds using SwissTargetPrediction yielded an initial set of 1,927 putative gene targets. Following the removal of redundant entries and harmonization to *Homo sapiens* gene symbols using the UniProt database, a final set of 658 unique protein targets associated with *A. Jacquemontii Blume* was established.

#### 7.1.2 The closeness analysis between the targets of *A. Jacquemontii Blume* and disease-associated targets

**FIGURE 2:**
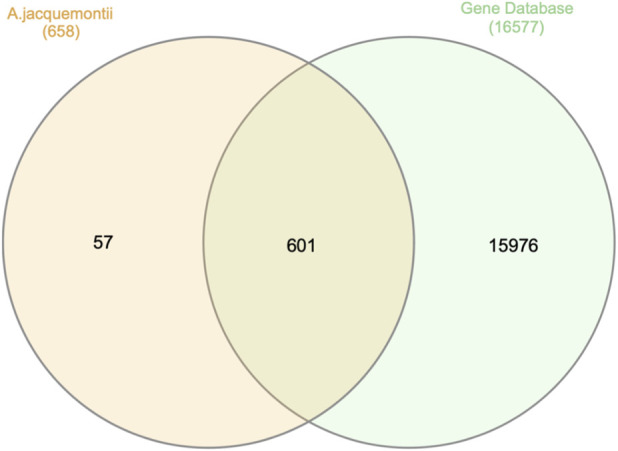
Venn diagram illustrating the intersection of gene targets identified for *A. Jacquemontii Blume* and CVD. The circle on the left illustrates the complete set of gene targets associated with *A. Jacquemontii Blume*, while The circle on the right denotes the total set of gene targets associated with atherosclerosis-related disease. The overlapping region indicates the shared gene targets between the two entities.

This study sought to explore therapeutic targets for atherosclerosis treatment using *A. Jacquemontii Blume*. To achieve this, leveraging publicly available databases like GeneCards and OMIM, this study established a broad collection of potential cardiovascular-associated gene targets. Upon removal of duplicate records, a combined total of 16,577 potential gene targets were curated from both sources.To mitigate the inclusion of irrelevant genes from this excessively large dataset and to achieve a more focused, target-driven selection, a Venn diagram analysis was performed. By intersecting the 16,577 disease genes with the 658 specific targets of the plant’s active ingredients, we eliminated the vast majority of off-target noise. This analysis revealed 601 highly specific potential target genes for *A. Jacquemontii Blume* in the context of atherosclerosis treatment. Figure 2 indicates the potential overlap.

#### 7.1.3 PPI network construction of common targets and Hub gene selection

Identification of potential therapeutic targets of *A. Jacquemontii Blume* for atherosclerosis was achieved through the construction and analysis of a protein interaction map or protein-protein interaction (PPI) network. An initial set of 601 putative target genes from *A. Jacquemontii Blume* was curated and meticulously subjected to PPI network construction harnessing the STRING database platform, employing a high-confidence minima interaction score threshold of >0.9. The resulting network was composed of 463 nodes and 1932 edges. While this represents a large interactome, it served strictly as the intermediate structural framework required for accurate topological calculations rather than the final gene selection. The PPI network is represented in Supplementary Figure 1.

To ensure the gene list was sufficiently filtered and highly specific to the disease pathology, these 463 nodes were subjected to a rigorous, two-step topological filtering pipeline. First, the network was visualized and analyzed using Cytoscape and the CytoNCA plugin. By employing five centrality measures betweenness, closeness, degree, eigenvector, and subgraph centrality we established a stringent selection criterion where only genes exhibiting values exceeding the median across all five metrics were retained. This strict filtering step successfully distilled the initial 463 nodes down to 68 key genes.

**FIGURE 3:**
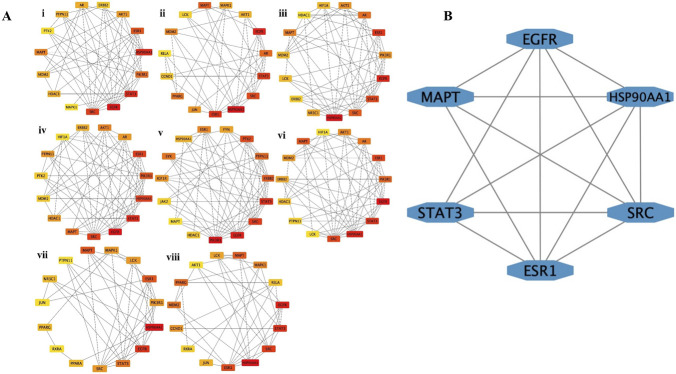
Identification of key hub genes from the PPI network. **(A)** Hub gene ranking based on eight CytoHubba topological algorithms: (i) Degree, (ii) Stress, (iii) Radiality, (iv) MNC, (v) MCC, (vi) Closeness, (vii) Bottleneck, and (viii) Betweenness. **(B)** Subnetwork highlighting the six consistently identified hub proteins (EGFR, MAPT, HSP90AA1, SRC, STAT3, ESR1) and their interactions within the PPI network.

To further refine this filtered list and identify the most critical regulatory proteins within the complex PPI network, an extensive centrality analysis was performed using another plugin of Cytoscape, CytoHubba. CytoHubba predicts and explores the most important nodes in a network based on several topological algorithms. In this study, we used eight algorithms: Degree, Stress, Maximum Clique Centrality (MCC), Betweenness, Bottleneck, Closeness, Radiality, and Markov Normalized Cumulative Flow (MNC) (see Figure 3A). To ensure a transparent and rigorous selection process, the top 10 highest-ranking genes from each of the eight independent algorithms were extracted and intersected. This strict overlap analysis consistently identified six consensus hub genes that were present across all eight distinct algorithmic assessments (see Figure 3B) These genes: HSP90AA1, ESR1, EGFR, SRC, STAT3, and MAPT potentially serve critical functions in cellular processes implicated in atherosclerosis.

For example, HSP90AA1 plays a vital role in controlling autophagy (Xiao et al., 2018), describes this as a key process vital for preserving cellular balance and ensuring survival in stress conditions that are frequently associated with atherosclerosis. While ESR1 functions in the regulation of diverse gene expression which participates in lipid metabolism, support vascular health, and modulates inflammation, positioning it as a promising target therapies addressing lipid associated atherosclerosis (Raina et al., 2020). The STAT3 primarily controls cell growth, division, and apoptosis (Akira, 2000) and its dysregulation is associated with chronic inflammation and fibrosis in atherosclerosis. The SRC gene functions as a non-receptor protein tyrosine kinase involved in signaling pathways that regulate cellular processes like differentiation, motility, adhesion and proliferation (Bjorge et al., 2000), these processes are critical for vascular remodeling and atherosclerosis. Additionally, the EGFR is involved in cell growth, survival, and angiogenesis, processes that are dysregulated in cardiovascular disease, and finally the MAPT is responsible for encoding tau protein (Zhang et al., 2016), which has been traditionally linked to neurodegenerative diseases. Emerging research suggest that tau could be involved in controlling vascular smooth muscle cells under stress conditions, thereby highlighting its relevance to the pathophysiology of atherosclerosis.

The identification of these six hub genes offers important insights into the intricate molecular mechanism underlying atherosclerosis. Moreover, these genes are not only relevant to atherosclerosis but are also implicated in other diseases, highlighting their potential as multi-disease therapeutic targets. Their involvement across various biological functions highlights their potential as key targets for future therapeutic development in atherosclerosis and other diseases. These six targets collectively underscore the multifactorial nature of atherosclerosis, where inflammation, oxidative stress, lipid metabolism, and vascular repair converge. This highlights the potential of *A. Jacquemontii Blume* bioactive compounds exert multi-target therapeutic effects, addressing several aspects of atherosclerosis pathology simultaneously.

#### 7.1.4 Construction of target-organ location

Understanding how various organs respond to diseases is crucial for developing better diagnostic and treatment strategies for complex diseases like atherosclerosis. Using microarray analysis of mRNA expression data from the BioGPS database, we mapped the six consensus hub genes (HSP90AA1, ESR1, EGFR, SRC, STAT3, and MAPT) identified from the network pharmacology analysis onto tissue-specific expression profiles across 84 organs and tissues. This integrative approach enabled the evaluation of the biological relevance of network-derived targets in organ-specific contexts. Comparative analysis of expression patterns revealed distinct tissue distribution profiles, which were further analyzed through network-based approaches to delineate the organ-specific roles of these targets. The analysis revealed that five genes exhibited significantly heightened expression in the pineal gland and CD34^+^ tissues, linking them to melatonin signaling and vascular health. Four genes indicated increased expression in metabolic organs such as the liver, prostate, and lungs, highlighting their potential roles in systemic metabolic regulation. Three genes were upregulated in the heart, smooth muscle, cardiac myocytes, and 721-B lymphoblasts, underscoring their involvement in cardiovascular and immune system regulation and two genes were predominantly expressed in myeloid cell lineages, including CD33^+^ and CD56^+^ cells, suggesting their role in inflammatory and immune responses.

The findings on the pineal gland align with the evidence supporting melatonin’s role in cardiovascular health. Melatonin, a hormone secreted by the pineal gland, is vital in mitigating oxidative stress and modulating inflammatory responses, key factors in preserving cardiovascular health (Możdżan et al., 2014; Nduhirabandi and Maarman, 2018; Zhao et al., 2021). Clinical research have demonstrated that melatonin supplements can improve left ventricular function, particularly in heart failure patients (Garakyaraghi, 2026). Additionally, administering melatonin acutely has been associated with a lower incidence of delirium in early stage heart failure cases (Yin et al., 2022), further emphasizing its protective cardiovascular effects. The study also highlights the dual role of CD34^+^ cells in vascular health. These cells support the regeneration of endothelial cells, which are essential for maintaining vascular integrity and repair. However, CD34^+^ cells are also involved in inflammatory processes, which can contribute to vascular diseases. This dual functionality suggests a therapeutic window for targeting CD34^+^ cells to enhance vascular repair while mitigating their pro-inflammatory effects, offering new avenues for treating vascular diseases.

**FIGURE 4:**
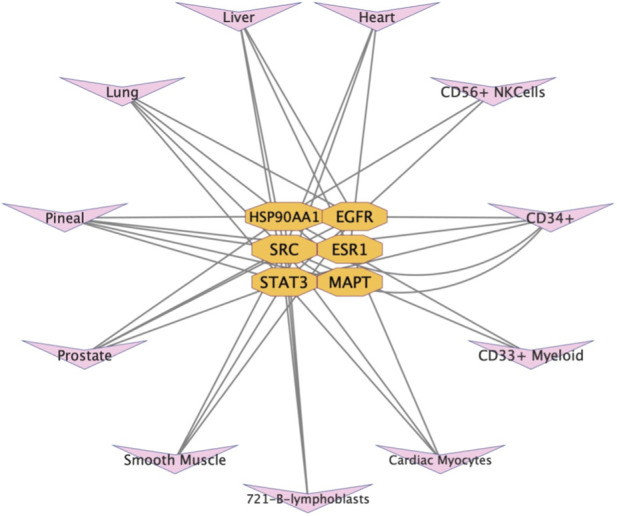
This network visualization illustrates the tissue distribution patterns of six key target genes across various human organs. Gene expression microarray data from BioGPS was used to identify organ-specific expression levels. Orange ellipse nodes represent the target genes, while pink arrow nodes indicate organ locations. The network highlights potential organ-specific effects of the target genes.

This evidence suggests that *A. Jacquemontii Blume* might exert beneficial effects on atherosclerosis by influencing melatonin production and signaling through the pineal gland, as well as by modulating CD34^+^ cell activity to support vascular repair and reduce inflammation. Melatonin has the ability to regulate oxidative stress and inflammatory responses further supporting its therapeutic potential in improving cardiovascular health. Apart from melatonin and CD34^+^ cells, STAT3, SRC, and ESR1 are three key genes that were expressed in the heart, vascular smooth muscle, and cardiac myocytes, emphasizing their roles in oxidative stress regulation, vascular remodeling, and lipid metabolism, respectively. Additionally, the tissue-specific expression patterns of other target organs (e.g., liver, prostate, lungs) and immune-related tissues underscore the multi-systemic nature of *A. Jacquemontii Blume* potential therapeutic effects. These findings highlight the complex interplay between organ systems in the pathophysiology of atherosclerosis (Figure 4).

#### 7.1.5 GO and KEGG analysis of Hub genes

For an in-depth examination of the biological processes and pathways associated with atherosclerosis, we conducted a comprehensive enrichment analysis on the six target genes identified from *A. Jacquemontii Blume*. This analysis utilized g:Profiler and Metascape to uncover fundamental mechanisms of atherosclerosis and identify potential therapeutic targets. First, we uploaded the hub gene information to g:Profiler for gene ontology (GO) analysis, resulting in a total of 211 GO entries, were identified and then classified into cellular components (CC) (14), biological processes (BP) (102), KEGG pathways and molecular functions (MF) (20) (11). Additionally, we also retrieve 28 Reactome genes and 32 WikiPathways gene sets. To enhance clarity and conciseness, the top five entries from each analysis category were selected for visualization.

The six hub genes identified in this study were primarily enriched in biological processes associated with inflammatory responses, hydrolase activity regulation, positive modulation of molecular function, stress response regulation, and defense responses. Notably, HSP90AA1 did not exhibit involvement in inflammatory responses or regulation of hydrolase activity. Previous reports indicate that individuals with a disruptive ESR1 mutation exhibit vascular dysfunction and suffer from early atherosclerosis. Activating Erα has been shown to lower infarct size, cardiomyocyte apoptosis, oxidative stress and inflammation, while inducing vasodilation and enhancing increasing vascular growth (Puzianowska-Kuźnicka, 2012). The genes ESR1, STAT3, EGFR, and SRC were linked to the regulation of hydrolase activity. Research indicates that endothelial cells exposed to simvastatin trigger phosphorylation of Akt, AMPK, and eNOS, thereby maintaining nitric oxide (NO) production. This process is accompanied by an increase in SHP phosphatase activity, relies on the activation of the EGFR-c-Src (Hou et al., 2015). Additionally, upon exposure to simvastatin, sEH undergoes rapid tyrosine phosphorylation, leading to enhanced interaction with Akt, AMPK, and eNOS, ultimately resulting in increased eNOS activation. Furthermore, the HSP90AA1 gene exhibits upregulated mRNA expression under stress conditions, highlighting its critical role in cardioprotection against ischemic injury. HSP90 is also integral to the PI3K-Akt survival pathway, which governs key cellular processes, including apoptosis prevention and endothelial function maintenance (Ranek et al., 2018; Zhu et al., 2016). Notably, previous studies (Cordeiro, 2025) have demonstrated that EGFR gene expression is significantly upregulated in animal models subjected to stress, further emphasizing its role in cardiovascular pathophysiology.

Enriched molecular functions primarily encompassed nitric oxide synthase regulation, protein kinase and phosphatase interactions, and ATP binding activities. The nitric-oxide synthase activity was particularly enriched in gene HSP90AA1, ESR1, and EGFR. The HSP90 plays a pivotal role in regulating nitric oxide production by modulating nitric oxide synthase (NOS) isoforms and cardiac oxygen metabolism through NOS regulation. These findings underscore the complex interplay between HSP90 and cardiovascular function (Presley et al., 2006). EGFR inhibition has been shown to attenuate NOX activity in diabetic models, this involves activation of the EGFR/ErbB receptor followed by oxidative stress through NOX which contribute significantly to vascular dysfunction in diabetes (Belmadani et al., 2008; Choi et al., 2012; Kassan et al., 2015). The protein kinase binding activity and kinase binding activity were enriched in all the hub genes. It has been reported that interaction of protein kinase C ζ and STAT3 results in cardiomyocyte hypertrophy (Li et al., 2016). Additionally, EGFR dimerization induces autophosphorylation of tyrosine residues within its intracellular kinase domain. This activation triggers downstream signaling cascades, including the MAPK pathway, through the recruitment of adaptor proteins (Holbro and Hynes, 2004).

The enriched CC is mainly related to the cell projection membrane, dendritic growth cone, growth cone, membrane raft, and membrane microdomain. The cell projection membrane was enriched in HSP90AA1, EGFR, and MAPT. The membrane raft term was found to be enriched in HS90AA1, EGFR, SRC, and MAPT. HSP90AA1 interaction with STAT3 and caveolin-1 in plasma membrane rafts has been reported to help prevent cytokine signaling during fever (Shah et al., 2002), Furthermore, caveolae and rafts play a regulatory role in the renal functions of vascular endothelial cells, tubular epithelial cells, and the nephron (Danielsen and Hansen, 2006). The endothelial cell Cav-1 in the membrane/lipid raft (MLR) is involved in regulating cell polarity and barrier functions (Hu et al., 2008). Prior findings have indicated the involvement of membrane rafts in EGRF, occurs largely via cholesterol disruption (Chen and Resh, 2002). A report identified the heterodimer of HER2 and EGFR as a key factor in the regulation switch by cholesterol and membrane rafts (Abousawan et al., 2022).

**FIGURE 5:**
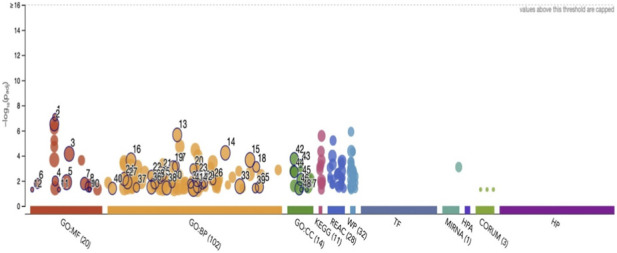
The hub genes functional enrichment analysis via g:Profiler is displayed, with Gene Ontology terms are color-coded as red for Molecular Function (MF), orange for Biological Process (BP), and green for Cellular Component (CC). Pathway enrichment results from KEGG, Reactome, and WikiPathways are represented by pink, purple, and blue colors, respectively.

The KEGG analysis uncovered pathways related to chemical carcinogenesis, estrogen signaling pathway, cancer proteoglycans, EGFR tyrosine kinase inhibitor resistance, and lipid metabolism and atherosclerosis, with gene involvement from HSP90AA1, ESR1, STAT3, EGFR, SRC, and MAPT. The REAC database was specifically enriched for signaling diseases involving signal transduction by growth factors, ERBB2, ESR-mediated signaling, signaling by ERBB4, and PI5P, PP2A, and IER3 regulates PI3K/AKT signaling. The WP database highlights pathways related to the aryl hydrocarbon receptor, nuclear receptors, relationship between inflammation COX2 and EGFR, microtubule cytoskeleton regulation, and EGFR tyrosine kinase inhibitor resistance. A detailed presentation of the enrichment analysis results is provided in Figure 5; Table 1. Subsequent verification of the g:Profiler findings was conducted using Metascape software. The validation findings are presented visually in Supplementary Figure 2, with corresponding annotations provided in Supplementary Table 2. Given the strong concordance between the g:Profiler and Metascape results, the identified mechanisms and pathways are likely to be accurate and pivotal in understanding the therapeutic effects of *A. Jacquemontii Blume* in atherosclerosis treatment.

**TABLE 1:** Results of functional enrichment analysis using g:Profiler.

Go term and pathways	Terms name	Term ID	Padj
Biological process	Regulation of hydrolase activity	GO:0051336	1.005 × 10^−4^
	Inflammatory response	GO:0006954	4.875 × 10^−4^
	Positive regulation of molecular function	GO:0044093	1.775 × 10^−5^
	Regulation of response to stress	GO:0080134	5.911 × 10^−5^
	Defense response	GO:0006952	3.380 × 10^−4^
Cellular components	Cell projection membrane	GO:0030426	1.781 × 10^−4^
	Membrane raft	GO:0045121	7.906 × 10^−3^
	Membrane microdomain	GO:0098857	7.988 × 10^−3^
	Growth cone	GO:0030426	1.589 × 10^−3^
	Dendritic growth cone	GO:0044294	4.063 × 10^−4^
Molecular functions	Protein kinase binding	GO:0019901	2.995 × 10^−7^
	ATPase binding	GO:0051117	2.311 × 10^−4^
	Nitric-oxide synthase regulator activity	GO:0030235	8.561 × 10^−8^
	ATPase binding	GO:0051117	2.311 × 10^−4^
	Protein phosphatase binding	GO:0019903	6.000 × 10^−6^
KEGG	Chemical carcinogenesis- receptor activation	KEGG:05207	2.617 × 10^−6^
	Estrogen signaling pathway	KEGG:04915	4.532 × 10^−5^
	EGFR tyrosine kinase inhibitor resistance	KEGG:01521	7.468 × 10^−4^
	Lipid and atherosclerosis	KEGG:05417	1.467 × 10^−4^
	Proteoglycans in cancer	KEGG:05205	2.344 × 10^−4^
REAC	Disease of signal transduction by growth factor receptor and second messengers	REAC:R-HSA-5663202	1.043 × 10^−4^
	Signaling by ERBB2	REAC:R-HAS-1227986	5.047 × 10^−4^
	PI5P, PP2A and IER3 regulate PI3K/AKT signaling	REAC:R-HSA-6811558	2.535 × 10^−3^
	Signaling by ERBB4	REAC:R-HSA-1236394	5.047 × 10^−4^
	ESR-mediated signaling	REAC:R-HSA-8939211	4.864 × 10^−4^
WP	Aryl hydrocarbon receptor pathway	WP:WP2586	1.220 × 10^−6^
	EGFR tyrosine kinase inhibitor resistance	WP:WP4806	1.940 × 10^−3^
	Relationship between inflammation COX2 and EGFR	WP:WP4483	4.758 × 10^−5^
	Nuclear receptors meta pathways	WP:WP2882	4.276 × 10^−5^
	Microtubule cytoskeleton regulation	WP:WP2038	3.122 × 10^−4^

## 8 Detection of genes with differential expression

**FIGURE 6:**
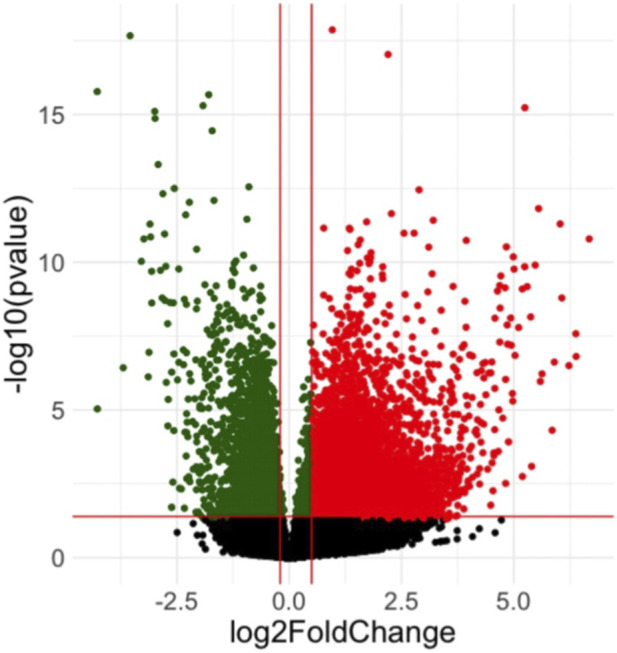
Volcano plot illustrating DEGs contrasting healthy and diseased cardiac tissues, with red markers signifying upregulated genes and green dots denote downregulated genes.

To discover genes associated with pulmonary hypertension caused by left heart disease, we analyzed gene expression data from the GSE23625. This dataset included samples from eight healthy hearts and fifteen diseased hearts. After thorough data preprocessing such as background correction and normalization, 7,634 differentially expressed genes (DEGs) were detected via a log2 fold change (L2FC) threshold of >0.5 and an adjusted p-value threshold of <0.05. Of these, 4,757 genes were upregulation, while 2,926 genes were downregulated. The distributions of DEGs, along with their statistical significance, are illustrated in the volcano plot (Figure 6).

### 8.1 Gene set enrichment analysis (GSEA)

**FIGURE 7:**
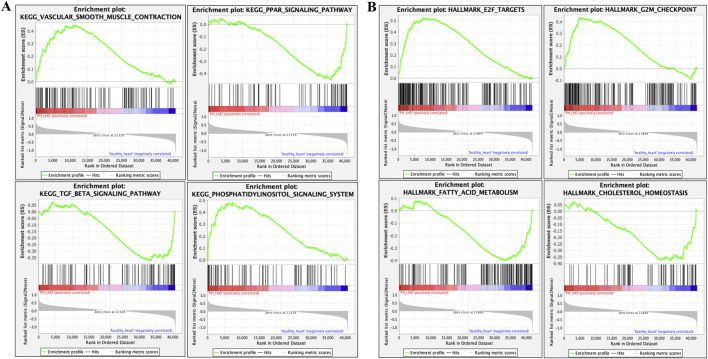
The image illustrates the results generated by GSEA software. **(A)** shows the KEGG pathway enrichment analysis for the PH-LHD group compared to the healthy heart group. **(B)** presents the hallmark gene set enrichment analysis, highlighting the differences between the PH-LHD and healthy heart conditions.

For a better understanding of gene expression patterns in GSE236251, Gene Set Enrichment Analysis (GSEA) was conducted. The study analyzed the DEGs identified between PH-LHD and the healthy heart group. Enrichment was evaluated using the KEGG pathways and Hallmark gene set (MSigDB) database, with a statistical significance defined as adjusted p-value <0.05 and FDR <0.25. Analysis against the KEGG pathways revealed that 114 gene sets were upregulated in the PH-LHD group. We observed significant enrichment in the vascular smooth muscle cell contraction pathway, suggesting its role in increased vascular tone and remodeling, hallmark features of severe vascular disease. Additionally, the phosphatidylinositol signaling system was also enriched, implicating dysregulated intracellular calcium signaling, which drives vascular smooth muscle hypercontractility. Conversely, protective pathways such as PPAR signaling pathway and TGF-beta signaling pathway (Figure 7A), were significantly downregulated in the diseased state and enriched in the healthy heart group, highlighting their critical role in regulating fatty acid metabolism and cardiac fibrosis to maintain normal vascular function.

Further analysis using the Hallmark gene set identified 16 gene sets upregulated in the PH-LHD group. Notably, E2F targets and the G2M checkpoint exhibited the highest enrichment scores, indicating dysregulated cell cycle progression and excessive proliferation of vascular smooth muscle cells. In contrast, the healthy heart group indicated upregulation of 34 gene sets, with significant enrichment in fatty acid metabolism and cholesterol homeostasis (Figure 7B), underscoring the vital role of energy homeostasis and lipid regulation in cardiovascular health. In summary, the data highlight the significant impact of disrupted vascular remodeling, cellular proliferation, and bioenergetic dysfunction in PH-LHD. Crucially, these specific pathological mechanism particularly vascular smooth muscle proliferation, endothelial dysfunction, and impaired cholesterol homeostasis, are also fundamental drivers of atherosclerosis.

### 8.2 Transcriptional factor analysis

**FIGURE 8:**
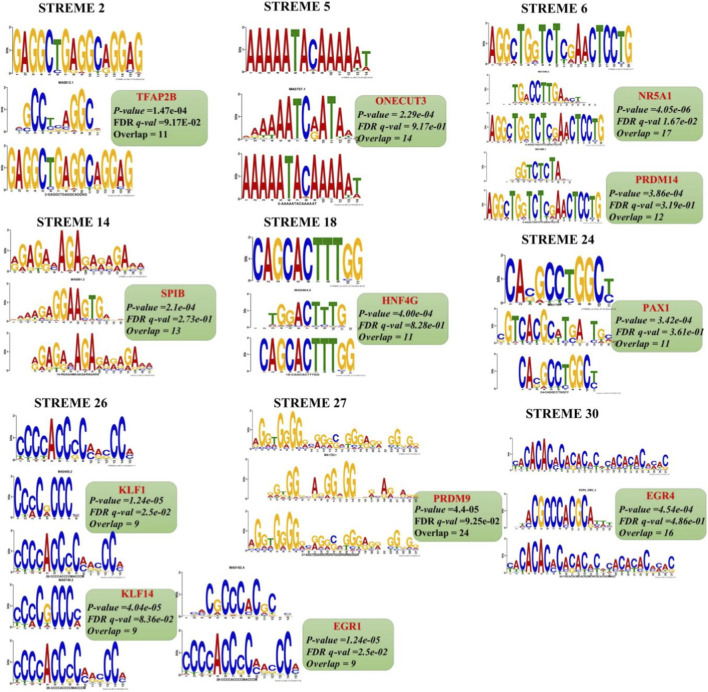
The figure presents *de novo* transcription factor (TF) motifs identified using the MEME algorithm. Motifs with statistically significant p-values and q-values are displayed, while those exhibiting similarity to known motifs, as determined by Tomtom analysis, are indicated.

Transcriptional factors (TFs) have been implicated in the pathogenesis of atherosclerosis by regulating epigenetic gene expression and influencing key morphogenetic processes (Duddu et al., 2020; Hu et al., 2024). Given this, we hypothesized that specific TFs might contribute to atherosclerosis progression by regulating cardiovascular-related genes. We used STREME tool, followed by Tomtom, to identify transcription factors interacting with the DEGs (see Figure 8). Our analysis identified several *de novo* TF motifs with significant overlap, including TFAP2B, ONECUT3, NR5A1, PRDM14, SPIB, HNF4G, PAX1, KLF1, KLF14, EGR1, PRDM9, and EGR4. Specifically, STREME2 overlaps with TFAP2B, STREME5 with ONECUT3, STREME6 with NR5A1 and PRDM14, STREME14 with SPIB, STREME18 with HNF4G, STREME24 with PAX1, STREME26 with KLF1, KLF14, and EGR1, STREME27 with PRDM9, and STREME30 with EGR4. All identified STREME motifs were upregulated in the DEGs, supporting their regulatory role in transcription during atherosclerosis progression.

Further examination with the MAST tool revealed that STREME2 is highly enriched in SCAND3, PLAAT1, TFAP2B, and MARCO. The MARCO gene is primarily expressed in macrophages but is also present in platelets, endothelial cells and vascular smooth muscle cells (Cavallari et al., 2018; Grolleau et al., 2003), playing a role in pathogen clearance and potentially in atherosclerosis, though its involvement in this context has been less studied. STREME5 overlaps with ONECUT3, whose promoter regions were observed in the CTSH and CD177 genes. CD177, an immune-related membrane glycoprotein, is upregulated in patients with severe infections and inflammation and correlates with increased neutrophil production (Lalezari et al., 1971; Wu et al., 2016), relevant to atherosclerosis progression. STREME6 overlaps with NR5A1 and PRDM14, with its promoter region observed in the RLP9 gene. Studies have shown that ribosome dysfunction, as seen with RLP9, is implicated in various atherosclerosis, particularly myocardial infarction (Fan et al., 2020; Taniguchi et al., 2002). STREME14 overlaps with SPIB, whose promoter region is found in the MCM2 gene, a gene widely studied in cancer research but also associated with cellular proliferation in other contexts, including CVD (Tran, 2020). STREME18 overlaps with HNF4G, which has been extensively implicated in hepatic lipid metabolism, though its precise role in cardiac development and function remains to be fully elucidated (Jiao et al., 2023) Finally, STREME26 overlaps with KLF1, KLF14, and EGR1, all of which play key roles in lipid metabolism, inflammation, and cellular proliferation. Notably, KLF14 has been specifically linked to the pathophysiology of atherosclerosis (Xie et al., 2017). In summary, the identified transcription factors, derived through STREME and validated using MAST, are integral to the multi-target therapeutic approach. They regulate key genes involved in lipid metabolism, inflammation, and cellular proliferation, all of which contribute to the progression of atherosclerosis. The findings indicate that focusing on these TFs could represent a novel strategy for addressing multiple pathways involved in atherosclerosis simultaneously, paving the way for future therapeutic interventions.

### 8.3 Gene regulatory network construction and analysis

**FIGURE 9:**
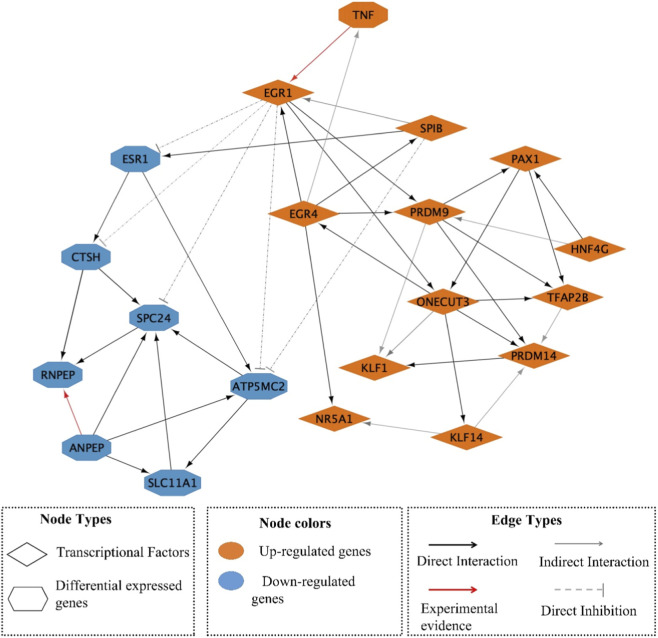
The gene regulatory network depicts interactions between transcription factors (TFs) and their target genes. Edges represent regulatory relationships, with color and line style indicating the nature of the interaction: Gray edge: Significant correlation based on either Spearman or Pearson coefficient. Denser edge: Significant correlation based on both Spearman and Pearson coefficients. Dotted edge: Negative correlation between TF and target gene. Red edge: Presence of experimental evidence supporting the interaction. The thickness of edges may represent the strength of the correlation or interaction, with thicker edges indicating stronger relationships.

To delineate the regulatory influence of DEGs in cardiovascular disease, the BNSA strategy was applied to construct gene regulatory networks (GRNs). By employing the splitting-average approach, we successfully generated a robust consensus network that minimized the false-positive interactions often inherent to transcriptomic datasets. Genes associated with the identified transcription factors (TFs) were selected using Spearman and Pearson correlation coefficient analysis of z-score normalized gene expression data, facilitating the construction of the gene regulatory network. Our analysis identified eight key genes SPC24, TNF, ATP5MC2, ESR1, ANPEP, RNPEP, CTSH, and SLC11A1, that exhibit direct and indirect interactions within the network Figure 9. Notably, tumor necrosis factor-alpha (TNF-α) and estrogen receptor 1 (ESR1) emerged as central hub genes. Under inflammatory conditions, elevated levels of TNF-α activate multiple downstream signaling pathways that regulate transcriptional responses associated with vascular inflammation. In our gene regulatory network analysis, TNF-α was observed to activate the transcription factor EGR1, consistent with experimental evidence demonstrating TNF-α-mediated induction of EGR1 during inflammatory stress (Jung et al., 2023). The interaction between TNF-α, EGR1, and c-Jun forms a regulatory complex that upregulates vascular endothelial growth factor receptor 2 (VEGFR2), thereby promoting angiogenesis. While angiogenesis is essential for endothelial repair, its dysregulation contributes to pathological processes such as atherosclerosis, diabetes, and cancer (Modi and Kulkarni, 2019). EGR1 activation further disrupts vascular health by binding to the ESR1 promoter and repressing its expression. This downregulation is pivotal, as ESR1 normally maintains endothelial homeostasis by promoting nitric oxide and suppressing NF-κB pathways. When ESR1 is inhibited, its protective “hub” functions fail, triggering a downstream cascade that reduces the expression of homeostatic genes like CTSH, RNPEP, SLC11A1, and SPC24. This shift creates a pro-inflammatory microenvironment characterized by lipid accumulation and oxidative stress, ultimately accelerating atherosclerotic plaque development. Interestingly, the network analysis also suggests that TNF-α-mediated signaling indirectly influences pathways linked to ESR1-associated protective responses, highlighting the complex interplay between inflammatory cytokines and hormonal signaling in cardiovascular disease. Additionally, the transcription factor SPIB indicated a direct interaction with ESR1, further supporting its central regulatory role within the network. Based on this mechanistic relationship, both TNF-α and ESR1 were selected as high-priority therapeutic targets for subsequent molecular docking and computational screening of phytochemicals derived from *A. Jacquemontii Blume*. Targeting these nodes offers a dual therapeutic strategy by inhibiting the pro-inflammatory cytokine TNF-α while simultaneously restoring or supporting the protective ESR1 signaling pathway.

Importantly, this GRN architecture clarifies the mechanism of action for the phytochemicals identified from *A. Jacquemontii Blume*. While these compounds do not dock directly to downstream transcription factors such as EGR1, TFAP2B, or KLF14, their robust inhibition of the upstream master regulators (TNF-α and ESR1) is predicted to disrupt these pathological signaling cascades. For instance, by suppressing TNF-α, the phytochemicals theoretically prevent the downstream activation of EGR1, thereby rescuing ESR1 from EGR1-mediated repression. By modulating the upstream mediators, these compounds exert indirect regulatory control over TF-mediated cellular proliferation, lipid metabolism, and inflammation.

### 8.4 Validation of docking protocol and molecular docking analysis of TNF-alpha and ESR1 with *A. Jacquemontii Blume* phytochemicals

**FIGURE 10:**
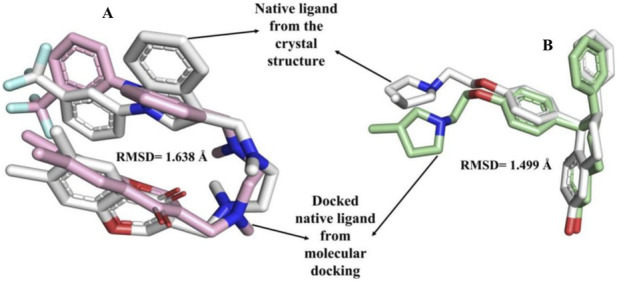
Superimposed structures of the native ligands (white) from the crystal structures and the re-docked ligands (pink and lime green) obtained from molecular docking calculations. The heavy-atom RMSD values were 1.638 Å for TNF-alpha **(A)** and 1.499 Å for ESR1 **(B)**, indicating accurate pose reproduction and successful validation of the docking protocol.

The reliability of the docking protocol was assessed through re-docking of native ligands into the active sites of the proteins. To assess the accuracy of the docking, the resulting docked poses were superimposed onto the original crystal structure poses (see Figure 10). Re-docking of the native ligand (307) into TNF-α yielded an RMSD of 1.638 Å, while ESR1 (native ligand R3V) showed an RMSD of 1.499 Å. Since both values are below the 2.0 Å threshold, the results indicate high-resolution docking and successful pose reproduction. By successfully reproducing the binding poses of these known standard compounds, we established a robust baseline to strictly benchmark the docking scores and binding interactions of the novel phytoconstituents. This validation supports that the selected parameters and algorithms are capable of accurately simulating natural binding interactions, thereby justifying the numerical methods used in this study (Ramírez and Caballero, 2018).

To support the targets identified through network pharmacology and gene regulatory network (GRN) inference, molecular docking was applied to analyze binding affinities and interaction energies of active compounds from *A. Jacquemontii Blume* with TNF-alpha and ESR1 proteins. TNF-alpha was prioritized due to its direct regulatory interaction with key transcription factors and its well-documented role in disease progression. Similarly, ESR1 was identified as a central hub gene and GRN component, highlighting its relevance as a therapeutic target. The docking studies were designed to clarify the binding mechanisms and examine the inhibitory potential of the compounds against TNF-alpha and ESR1. The computed binding free energies varied between −3.9 and −7.0 kcal/mol. Gamma-sitosterol had the strongest affinity for TNF-alpha at −7.0 kcal/mol, while its standard (native ligand) scored −5.7 kcal/mol (Supplementary Table 3). Phenol-derived compound showed the highest binding for ESR1-alpha with −6.7 kcal/mol, compared to its standard (native ligand) score of −6.0 kcal/mol (Supplementary Table 4). To further analyze these interactions, the top docked complex were visualized using Discovery Studio Biovia and PyMol, facilitating a detailed examination of the molecular interactions within the binding sites.

**FIGURE 11:**
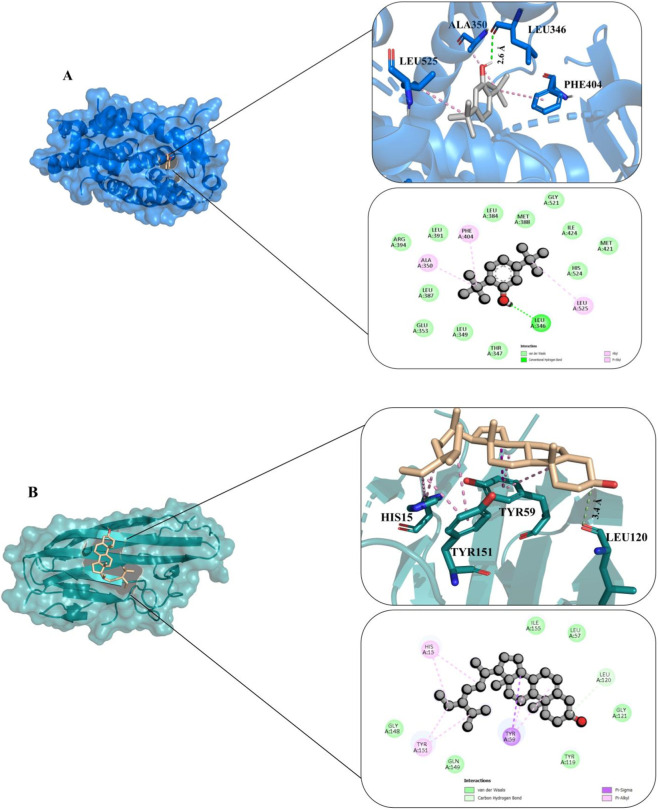
**(A)** The left panel illustrates the ESR1 protein in surface and cartoon representation, colored marine blue, with the ligand Phenol-derived compound shown in grey occupying the active site. The top-right inset provides a 3D close-up of the ligand conformation within the binding pocket, highlighting its spatial fit. The bottom-right panel presents a 2D schematic of the docking complex, displaying the ligand’s orientation and surrounding residues within the binding cavity. **(B)** The left panel illustrates the TNF-alpha protein in surface and cartoon representation (depicted in deep teal blue), with Gamma-sitosterol (wheat color) bound within the active site cavity. The top-right inset offers a close-up view of the ligand positioned in the binding pocket, highlighting the spatial arrangement and nearby amino acid residues. The bottom-right panel presents a 2D schematic of the docking complex, showing the ligand’s orientation relative to surrounding residues and the overall topology of the binding site.

Figure 11A illustrates the interaction between a phenol-derived phytochemical from *A. Jacquemontii Blume* and the Estrogen Receptor Alpha (ESR1) protein (PDB ID: 7UJW). Molecular docking highlights a favorable binding energy of −6.7 kcal/mol, indicating a strong interaction within the receptor active site. The compound is accommodated within a pocket comprising residues M343, L346, T347, A350, D351, R352, E353, L354, L387, M388, M391, R394, F404, M421, G521, H524, and L525, which span structurally and functionally important regions of the Ligand Binding Domain (LBD). The ligand interacts with twelve amino acid residues primarily via van der Waals forces (Gly521, Met388, Leu384, Leu391, Arg394, Leu387, Glu353, Ala350, Leu349, Thr347, His524, and Met421), forming a tightly packed hydrophobic pocket that ensures complex stability. Despite its relatively small size, the ligand forms a conventional hydrogen bond with Leu346 (2.6 Å), while alkyl interactions with Ala350 and Leu525 further enhance binding affinity. These interactions are essential for stabilizing the ligand’s conformation and facilitating strong molecular anchorage. The engagement with His524 and Leu525 is mechanically significant. Research suggests these specific residues act as a “selectivity switch” for the receptor; interactions here can shift binding preferences by two orders of magnitude and create steric hindrance that blocks natural ligands like estradiol. By anchoring to this critical region, the phytochemical influences the orientation of Helix 12, effectively preventing the protein from recruiting co-activators (Arévalo-Salina et al., 2025). This mechanism “turns off” pro-inflammatory gene expression, providing a clear pathway for how this compound reduces vascular inflammation in atherosclerosis. Collectively, these hydrophobic and polar interactions underscore the specificity and stability of the target-ligand complex, supporting its potential as a therapeutic lead.

Figure 11B illustrates the molecular docking interaction between the Tumor Necrosis Factor-alpha monomer (TNF-alpha; PDB ID: 2AZ5) and Gamma-sitosterol, a bioactive phytoconstituent derived from the rhizome of *A. Jacquemontii Blum*e. Among the screened compounds, Gamma-sitosterol showed the favorable binding affinity, with a docking score of −7.0 kcal/mol, indicating stable and favorable interactions at the monomer interface. Because the transition between the TNF-alpha monomer and its active trimer is a highly dynamic process, targeting the monomer interface is a strategic mechanism to prevent functional trimerization. The ligand is accommodated within a critical active-site region comprising residues L57, Y59, S60, Q61, Y119, L120, G121, G122, and Y151. Detailed interaction profiling revealed a π–σ interaction with Tyr59, a key interface residue contributing to conformational stabilization. Hydrophobic stability is further enhanced by π–alkyl interactions with His15 and Tyr151, alongside a carbon–hydrogen bond with Leu120 (3.4 Å). Furthermore, a dense network of van der Waals interactions involving Ile155, Gly148, Gln149, Tyr119, Gly121, and Leu57 reinforces the ligand’s orientation. By stably binding to these critical interface residues on the monomer, Gamma-sitosterol effectively masks the dimerization/trimerization interface, theoretically preventing the assembly of the biologically active TNF-alpha cytokine.

The favorable binding affinity and stability indicated by Gamma-sitosterol and the phenol-derived phytochemical highlight their potential as strong therapeutic candidates from *A. Jacquemontii Blume*. Specifically, *in silico* predictions suggest the phenol-derived compound modulates ESR1 to support lipid metabolism and vascular homeostasis, while Gamma-sitosterol acts as a theoretical TNF-alpha inhibitor by binding the monomer interface to prevent active trimerization. Overall, these computational findings indicate that these bioactive compounds hold significant potential for the development of multi-target pharmaceutical agents against atherosclerosis, warranting future experimental validation.

### 8.5 Molecular dynamic simulation

To gain deeper insight into the docking results and evaluate the conformational integrity and binding characteristics of promising compounds at the substrate-binding sites of TNF-alpha and ESR1 proteins, MD simulations were performed for the TNF-alpha–Gamma-sitosterol and ESR1–Phenol-derived complexes. To establish a clear benchmarking approach, these trajectories were strictly compared against their respective small-molecule reference standards (the native ligands). Each complex underwent a 300-nanosecond simulation. Structural parameters, including Root-Mean-Square Deviation (RMSD), Root-Mean-Square Fluctuation (RMSF), radius of gyration (Rg), and time-dependent hydrogen bond formation, were estimated to assess system stability. To ensure rigorous statistical validation, standard deviations for all structural parameters were calculated across the sampled trajectory frames. The average values and their corresponding standard deviations are summarized in Table 2.

**TABLE 2:** Average values of structural parameters for TNF–alpha-Gamma-sitosterol and ESR1–Phenol-derived complexes over the 300 ns MD simulation. The table presents the average values of RMSD, RMSF, radius of gyration, and H-bond for both protein–ligand complexes and their respective standard (native ligand), providing insights into their structural stability and compactness during the simulation period.

	TNF-alpha		ESR1	
MD Parameter	Gamma-sitosterol	Standard	Phenol-derived compound	Standard
RMSD	0.205 ± 0.03	0.231 ± 0.05	0.186 ± 0.019	0.207 ± 0.02
RMSF	0.111 ± 0.07	0.126 ± 0.06	0.094 ± 0.055	0.103 ± 0.065
H-BOND	0.212 ± 0.66	0.014 ± 0.12	0.424 ± 0.60	0.305 ± 0.483
RG	1.540 ± 0.007	1.539 ± 0.01	1.741 ± 0.007	1.742 ± 0.009

#### 8.5.1 Stability analysis of TNF-alpha-gamma-sitosterol

**FIGURE 12:**
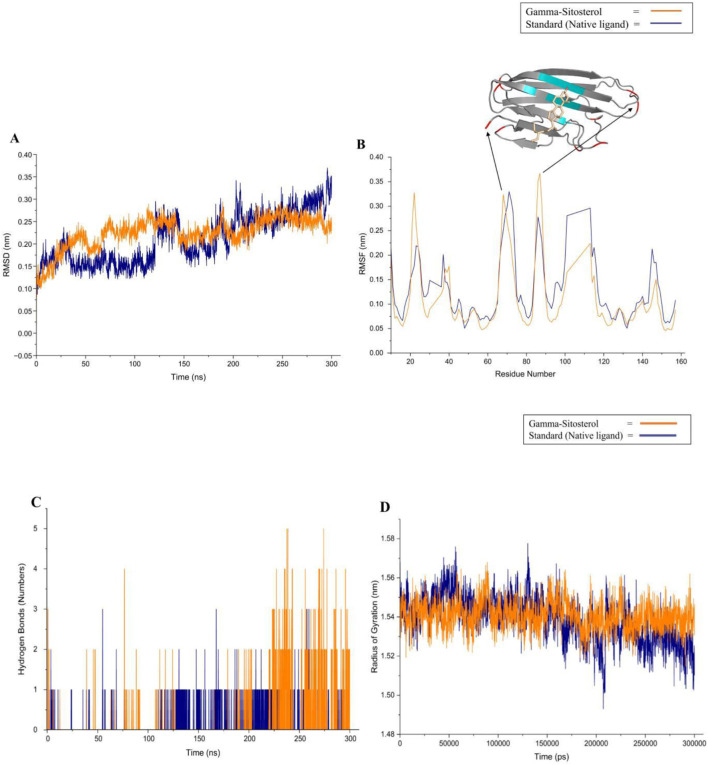
Molecular dynamics analysis of the TNF-alpha receptor in complex with Gamma-sitosterol and the native ligand (standard). **(A)** Root Mean Square Deviation (RMSD) plot depicting backbone stability of TNF-alpha bound to Gamma-sitosterol (orange) and the standard ligand (blue) over a 300 ns simulation period. **(B)** Root Mean Square Fluctuation (RMSF) plot showing residue-wise flexibility of TNF-alpha for the Gamma-sitosterol complex (orange) and the standard complex (blue). The inset highlights the TNF-alpha–Gamma-sitosterol binding mode, with loop regions exhibiting increased fluctuation (red) and the stabilized active site residues (teal). The bound Gamma-sitosterol molecule is shown in stick representation. **(C)** Hydrogen bond formation and **(D)** radius of gyration of TNF-alpha bound to Gamma-sitosterol (orange) and the standard ligand (blue) over the 300 ns simulation.

In order to analyze the stability and dynamic behavior of the TNF-alpha–Gamma-sitosterol and TNF-alpha–standard (native ligand) complexes, molecular dynamics (MD) trajectories were analyzed over a 300-nanosecond simulation using root mean square deviation (RMSD). RMSD is a widely recognized measure of conformational shifts in protein–ligand complexes, offering critical insights into system stability, equilibrium, and binding effectiveness. As shown in Figure 12A, the TNF-alpha–Gamma-sitosterol complex (orange line) exhibited a relatively stable RMSD profile. After an initial equilibration period, the values gradually increased and stabilized, plateauing around 0.20 nm. This implies that the complex preserved its structural integrity with limited fluctuations over the course of the simulation. Conversely, the TNF-alpha complex benchmarked against the standard reference inhibitor (blue line) showed greater conformational variability, with RMSD values gradually increasing to approximately 0.30 nm by the end of the simulation, indicating increased backbone flexibility and less stable binding.

Quantitatively, the Gamma-sitosterol complex maintained an average RMSD of 0.205 ± 0.030 nm, while the standard ligand complex demonstrated comparatively higher values of 0.231 ± 0.050 nm (Table 2). Crucially, the phytochemical has a lower mean and a 40% lower standard deviation compared to the native ligand, indicating much higher structural reproducibility. These standard deviations provide statistical validation of the restricted conformational drift and enhanced structural integrity of the complex. The lower and more consistent RMSD range of the Gamma-sitosterol-bound complex reflects reduced conformational drift and enhanced structural integrity, suggesting more stable and sustained interactions with the TNF-alpha binding site. Notably, the TNF-alpha complex bound to Gamma-sitosterol, an active phytochemical isolated from Arisaema jacquemontii Blume, displayed a distinct and favorable stability profile compared to the native ligand. Its narrower deviation and consistent conformational behavior highlight its potential to engage TNF-alpha more effectively and persistently. Collectively, these results indicate that Gamma-sitosterol forms a more stable and enduring interaction with TNF-alpha than the standard ligand. The reduced RMSD and conformational rigidity of the complex suggest stronger binding affinity and improved inhibitory potential, reinforcing Gamma-sitosterol promise as a natural therapeutic candidate for modulating TNF-alpha mediated inflammatory responses.

We delved even further into the protein-ligand interactions by analyzing the Root Mean Square Fluctuation (RMSF) values. This metric provides a detailed picture of how much individual amino acids (residues) in the protein fluctuate during the 300-nanosecond simulation for both the TNF-alpha-Gamma-sitosterol and TNF-alpha–Standard complexes. The RMSF profile, presented in Figure 12B, highlights the residue-wise fluctuations, offering insights into the local mobility and conformational adaptability of protein regions upon ligand binding. The orange curve, representing Gamma-sitosterol, shows slightly reduced overall fluctuations compared to the blue curve of standard ligand. The average RMSF was 0.111 ± 0.07 nm for the Gamma-sitosterol complex and 0.126 ± 0.06 nm for the standard, providing robust statistical validation that the Gamma-sitosterol complex maintains a relatively more stable conformation, particularly in regions proximal to the binding site. Residue-level analysis identified five residues with notable fluctuation (>0.2 nm) in the Gamma-sitosterol-bound complex: Asp10, Gln21, Gly68, Glu86, and Pro113. In contrast, the standard complex exhibited fluctuations in eight residues: Asp10, Ala22, Leu37, Ser71, Val85, Cys101, Pro113 and Ala142. The overlap of Asp10 and Pro113 in the Gamma-sitosterol-bound complex indicates that these regions are inherently flexible or play a role in local structural adjustments during ligand accommodation. The structural inset in Figure 12B depicts the protein backbone and highlights fluctuation prone regions upon Gamma-sitosterol binding. Red colored segments correspond to loop regions exhibiting pronounced flexibility, which is typical for loops as they often adapt to support ligand interaction. Notably, the teal colored regions indicate the active site residues, which display minimal fluctuation, statistically validating that the active site maintains strict conformational stability in the presence of Gamma-sitosterol. The localized flexibility remains primarily within peripheral loops, supporting the notion that Gamma-sitosterol binding preserves the functional integrity of the catalytic region while allowing adaptable movement in non-critical regions. Collectively, these observations demonstrate that Gamma-sitosterol stabilizes the TNF-alpha active site while permitting loop flexibility, a dynamic balance characteristic of effective protein–ligand engagement (Figure 12B).

To further elucidate the interaction strength and binding stability of Gamma-sitosterol with TNF-alpha, a comprehensive hydrogen bond analysis was performed over the 300 ns molecular dynamics (MD) simulation trajectory (Figure 12C). Hydrogen bonding serves as a fundamental non-covalent interaction that significantly contributes to ligand specificity, retention, and the overall structural stability of protein–ligand complexes. The hydrogen bond formation profile elucidated distinct interaction patterns between TNF-alpha and its two ligands Gamma-sitosterol (orange line) and the standard reference compound (blue line). The TNF-alpha–Gamma-sitosterol complex consistently exhibited the formation of multiple hydrogen bonds, particularly showing increased bonding frequency and intensity beyond 200 ns, with peaks reaching up to five hydrogen bonds during specific intervals. In stark contrast, the TNF-alpha–standard complex demonstrated a sparse and irregular hydrogen bonding pattern, with sporadic instances rarely exceeding 1–2 hydrogen bonds and large intervals devoid of any interactions. Quantitative analysis showed that Gamma-sitosterol formed an average of 0.212 ± 0.66 hydrogen bonds, while the standard ligand maintained a significantly lower average of 0.014 ± 0.12 hydrogen bonds throughout the simulation. The relatively higher standard deviation associated with Gamma-sitosterol reflects its capacity for dynamic and transient polar interactions across diverse binding site residues. This behavior suggests that while hydrogen bonding may not be continuously sustained, the ligand engages in recurrent, stabilizing interactions that contribute to its robust occupancy within the active site. Conversely, the minimal and unstable hydrogen bond profile of the standard ligand reflects a weaker, less persistent interaction landscape, indicative of reduced binding stability. These results collectively strengthen the case for Gamma-sitosterol as a promising natural inhibitor capable of modulating TNF-alpha activity with high specificity and sustained interaction (Figure 12C; Table 2).

To further evaluate the compactness of the TNF-alpha complexes, the radius of gyration (Figure 12D; Table 2) was analyzed for 300 ns MD simulation. The radius of gyration (Rg) reflects how atoms are spatially distributed around a protein center of mass, offering valuable information about the structural compactness and folding stability of protein–ligand complexes. The Rg profiles for both the TNF-alpha–Gamma-sitosterol complex (orange line) and the standard ligand complex (blue line) exhibit stable trends with only minor fluctuations throughout the simulation period. This indicates that both complexes maintain their compact, folded states under the simulated physiological conditions. Quantitatively, the average Rg for the TNF-alpha–Gamma-sitosterol complex was 1.540 ± 0.007 nm, which is nearly identical to the average Rg of the standard complex, 1.539 ± 0.01 nm. While the mean values are nearly identical, the remarkably lower standard deviation (0.007 nm) for Gamma-sitosterol provides robust statistical validation, proving that the complex remained more consistently compact than the standard throughout the trajectory. The small difference suggests that Gamma-sitosterol binding does not significantly alter the conformational dimensions of TNF-α relative to the standard ligand. As a result, the stable Rg values further support the findings from the RMSD and RMSF analyses, reinforcing that Gamma-sitosterol binding preserves the compactness and overall structural integrity of TNF-alpha throughout the simulation timeframe. This consistent compactness is indicative of a stable and well-maintained folded state, which is desirable for maintaining protein function and effective ligand binding.

#### 8.5.2 Stability analysis of ESR1- phenol -derived compound

**FIGURE 13:**
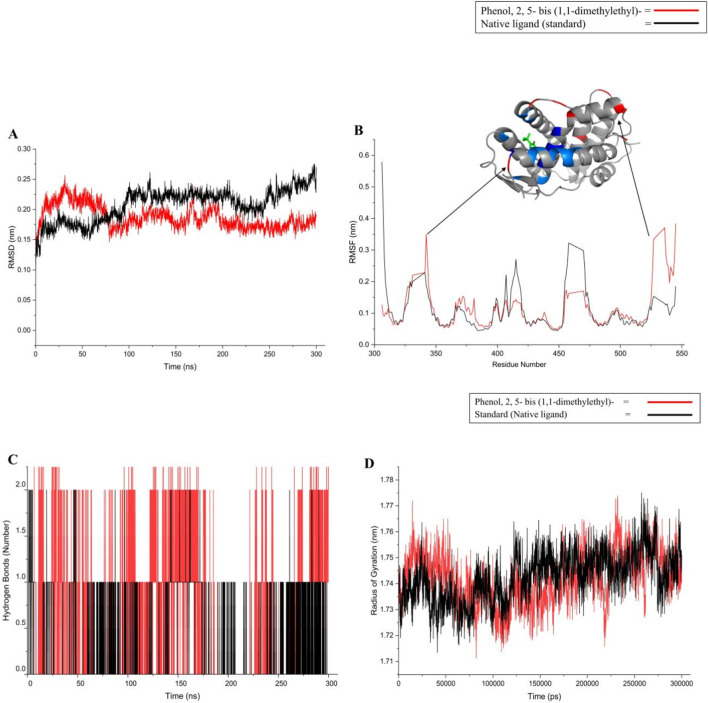
Molecular dynamics analysis of the ESR1 receptor bound to Phenol, 2,5-bis(1,1-dimethylethyl)- and the standard (native ligand). **(A)** Root Mean Square Deviation (RMSD) plot showing backbone stability of ESR1 in complex with Phenol-derived (red) and the standard (black) over a 300 ns simulation. **(B)** Root Mean Square Fluctuation (RMSF) plot illustrating residue-wise flexibility for ESR1–Phenol (red) and ESR1–standard (black) complexes. The inset depicts the ESR1–Phenol structure highlighting residues that exhibit increased fluctuations (red) and the active site residues (blue). The green stick model represents the bound Phenol ligand. **(C)** Hydrogen bond formation and **(D)** radius of gyration of ESR1 bound to Phenol-derived (red) and the standard ligand (black) over the 300 ns simulation.

The MD trajectories was performed to investigate the RMSD in order to ascertain the behavior of the Phenol-derived and ESR1-standard (native ligand) complexes throughout the 300-nanosecond simulation. The ESR1–Phenol complex (displayed by the red curve) exhibited a sharp increase in RMSD during the initial 50 ns, followed by a consistent plateau phase, indicating the system reached a stable conformation (Figure 13A). The average RMSD value for this complex was 0.186 ± 0.019 nm, suggesting minimal conformational drift and stable ligand accommodation within the active site. In contrast, the ESR1 complex bound to the established reference benchmark (shown in black) displayed relatively higher fluctuations, with an average RMSD of 0.207 ± 0.020 nm (Table 2), reflecting slightly greater conformational flexibility and less structural rigidity during the simulation. Interestingly, the ESR1 complex bound to the identified active ingredient of *Arisaema Jacquemontii Blume*, Phenol-derived compound, displayed a distinct stability profile compared to the standard ligand. Its lower RMSD range and narrower lowe standard deviation suggest a more favorable and stable interaction within the ESR1 binding pocket. Collectively, these results suggest that the identified phytochemical maintains a more stable interaction with ESR1 than the native ligand, supporting its potential as a promising candidate for therapeutic modulation of estrogen receptor activity.

To further evaluate the dynamic flexibility of the ESR1–ligand complexes at the residue level, Root Mean Square Fluctuation (RMSF) analysis was performed over the 300-nanosecond molecular dynamics simulation. RMSF quantifies the averaged positional deviation of individual amino acid residue from its mean position, thus providing insight into local motions within the protein structure upon ligand binding. As depicted in Figure 13B, the RMSF profiles of ESR1–Phenol-derived and ESR1–standard (native ligand) complexes are represented by red and black curves, respectively. The Phenol-bound complex exhibited lower overall fluctuations, exhibiting an average RMSF of 0.094 ± 0.055 nm, while the standard ligand complex demonstrated a sightly higher average fluctuation of 0.103 ± 0.060 nm (Table 2). These computed standard deviations serve as robust statistical validation of the stabilized local motions within the active site core. Furthermore, the lower standard deviation observed for the phytochemical complex statistically validates a more uniform and stable amino acid residue behavior throughout the protein backbone compared to the native ligand. Although the average RMSF values are close, the residue-specific analysis reveals highlights clear difference in the pattern and distribution of flexibility. Specifically, the Phenol-derived bound complex showed four with a marked fluctuations exceeding 0.2 nm.

These residues include: Leu306, Ser527, Lys536, and Asp545. These fluctuation-prone residues are visually highlighted in red within the protein structure inset (Figure 13B), confirming that phenol binding induces localized flexibility, while maintaining overall structural coherence. In contrast, the ESR1–standard ligand complex exhibited significant fluctuations in six distinct residues: Ser341, Ser329, Pro406, Gly415, Tyr459, and Glu470. These regions also predominantly correspond to flexible loops, yet their fluctuation patterns show a distinct spatial arrangement, potentially reflecting adaptive modes of protein response upon ligand engagement. Importantly, the blue colored regions in the structure denote the active site residues, which remained conformationally stable in the Phenol-bound complex. This suggests that Phenol binding does not compromise the structural integrity of ESR1 functional core. Notably, the observed fluctuations occur away from the active site, indicating that Phenol complex induces peripheral flexibility while preserving the catalytic center. The RMSF analysis thus underscores that the Phenol complex binds within the same active regions as the experimentally determined ligand, maintaining the receptor core structural stability while permitting necessary motion in non-critical loops. This dynamic behavior supports its potential to engage ESR1 effectively without destabilizing its functional architecture.

To obtain a more detailed understanding of the binding stability of the ESR1–ligand complexes, we analyzed the formation of hydrogen bonds throughout the 300-nanosecond molecular dynamics simulation. By supporting moleculare recognition and bolstering complex stability, hydrogen bonds are essential foe maintaining target-ligand interactions. Figure 13C presents the hydrogen bond time evolution profile for the ESR1–Phenol complex (red) and the ESR1–Standard complex (black). The hydrogen bond occupancy was evaluated over the entire simulation trajectory to assess the persistence and consistency of these interactions. The ESR1–Phenol complex demonstrated a higher average number of hydrogen bonds (0.424 ± 0.60) compared to the standard complex (0.305 ± 0.48) (Table 2), suggesting that the natural ligand forms more frequent and stable hydrogen bonding interactions with ESR1. These interactions are distributed intermittently throughout the simulation but show noticeable clusters of stability, particularly around the 50–100 ns, 140–180 ns, and 240–280 ns intervals.

In contrast, the standard ligand exhibited fewer and more sporadic hydrogen bonds, with limited periods of sustained interaction. This reduced hydrogen bond occupancy suggests weaker interaction affinity and a less stable complex. The enhanced hydrogen bonding capacity of Phenol-derived indicates a stronger and more consistent interaction with ESR1, which may contribute to improved binding stability and functional modulation of the receptor. Notably, the increased fluctuation observed in hydrogen bonding for Phenol also reflects its dynamic engagement with the binding pocket, allowing for adaptive interactions while maintaining overall complex integrity. Therefore, the findings affirm the superior interaction profile of Phenol relative to the standard ligand, strengthening its candidacy as a potential natural modulator of ESR1 activity.

To assess the compactness and conformational stability of the ESR1–bound ligand complexes over the course of the 300-nanosecond molecular dynamics simulation, we calculated the Radius of Gyration (Rg), It is a measure of how far atoms are, on average, from their shared center of mass, weighted by their mass. This metric serves as an important indicator of overall protein folding and tertiary structural integrity. Figure 13D presents the Rg profiles of the ESR1 complexes with Phenol-derived compound (red curve) and the standard ligand (black curve). Throughout the trajectory, both complexes exhibited consistent Rg values without significant deviation, indicating that the ESR1 protein remained structurally stable in both cases.

The average Rg for the ESR1–Phenol complex was 1.741 ± 0.007 nm, while that for the ESR1–Standard complex was 1.742 ± 0.009 nm (Table 2). While the mean values are nearly identical, the remarkably lower standard deviation (0.007 nm) for the phytochemical confirms that it held the protein in a more consistently compact state, providing robust statistical validation of its persistent folding stability over the entire 300-ns trajectory. The minimal difference between the two values suggests a comparable degree of global structural compactness across both systems. However, the slightly lower and more fluctuating Rg observed in the ESR1–Phenol complex during specific intervals (e.g., ∼60–100 ns and ∼160–200 ns) may indicate dynamic structural adaptations in response to ligand binding, while still maintaining overall fold stability. These subtle fluctuations could reflect ligand-induced local flexibility, particularly in peripheral or loop regions, without compromising the core architecture of the protein. Importantly, the absence of any abrupt Rg shifts throughout the simulation in both systems confirms the absence of unfolding events, further supporting the conformational resilience of the ESR1 receptor upon ligand binding. In summary, the Rg analysis confirms that both the natural ligand Phenol and the standard compound preserve the compactness and structural integrity of ESR1, with Phenol showing a slightly more dynamic yet stable interaction profile. This reinforces its potential as a reliable and effective modulator of ESR1 conformation.

Overall, these results show that both *Arisaema Jacquemontii Blume* bioactives exhibit strong multi-target binding, stabilizing TNF-alpha and ESR1 without disrupting their functional cores. The reduced RMSF in loop regions and stable active sites suggest maintained structural integrity and effective interaction. Furthermore, the rigorous statistical validation of these structural parameters highlighted by the consistently lower standard deviations observed for the selected phytochemicals compared to the native ligands confirms the high reproducibility of the simulations and statistically validates their superior capacity to minimize conformational fluctuations and maintain highly stable complexes. Therefore, the molecular dynamics evidence supports their potential as natural multi-target modulators for atherosclerosis and inflammatory diseases.

#### 8.5.3 Metadynamic analysis of the trajectories

##### 8.5.3.1 PCA and dynamic cross-correlation matrix

**FIGURE 14:**
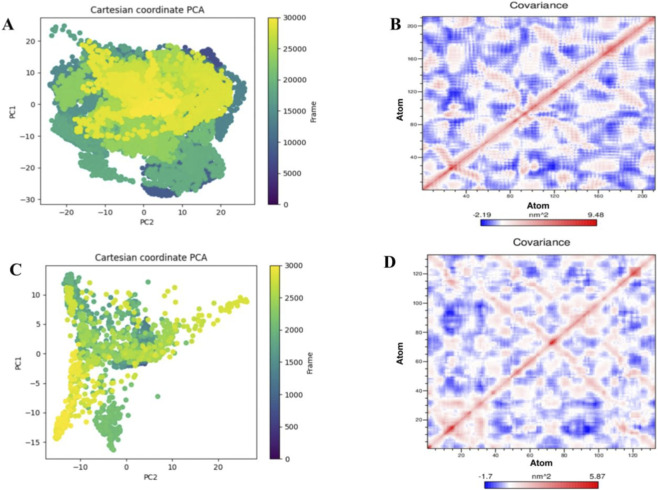
Principal Component Analysis (PCA) and Dynamic Cross-Correlation Matrix (DCCM) plots for ESR1–Phenol and TNF-alpha–Gamma-sitosterol complexes. **(A)** PCA plot (left) and **(B)** DCCM map (right) for the ESR1 receptor bound to Phenol, 2,5-bis(1,1-dimethylethyl)-, illustrating the dominant motions (PC1 vs. PC2) and residue correlation patterns. **(C)** PCA plot (left) and **(D)** DCCM map (right) for TNF-alpha bound to Gamma-sitosterol, showing principal component distribution and correlated (red) and anti-correlated (blue) atomic motions during the simulation.

To explore the conformational dynamics and flexibility of TNF-alpha and ESR1 upon ligand interaction, Principal component analysis (PCA) and Dynamic cross-correlation matrix (DCCM) analyses were carried out to investigate the system dynamics of both target proteins complexed with bioactive compounds from *Arisaema Jacquemontii Blume*, along with their respective standard ligands. The scatter plots in Figure 14A; Supplementary Figure 3A depict the dominant dynamic modes captured over the 300 ns simulation, with the ESR1 bound to Phenol-derived compound complex showing a well-dispersed yet organized spread characterized by multiple overlapping clusters along both PC1 and PC2. The accompanying color gradient from dark blue (early frames) to yellow (late frames) illustrates the progression of the simulation, indicating continuous structural transitions over time. This dynamic pattern, particularly the visible spread in the central and upper-right quadrants, suggests active conformational sampling and flexible adjustments of the receptor when bound to the Arisaema-derived ligand. In contrast, the ESR1–standard complex displays a tightly confined, nearly linear clustering along PC2 with minimal dispersion and a static color gradient, indicating that the receptor explores a much narrower conformational space in the presence of the standard ligand. This restricted motion reflects lower intrinsic flexibility and limited essential dynamics compared to the Phenol-bound form. To further characterize internal motion, DCCM plots were generated from the Cα atom trajectories over the 300 ns simulation period. The DCCM visualizes (Figure 14B) residue–residue correlated motions, where positive correlations (red) represent synchronized movements and negative correlations (blue) indicate opposing motions, with correlation values ranging from −1 to +1. The ESR1-Phenol derived complex displayed a well-defined pattern of both positive and negative correlations, particularly within the helical regions and the active site, indicating enhanced inter-residue communication and coordinated domain-level dynamics that help stabilize the ligand-binding pocket and preserve overall structural integrity. In contrast, the standard ligand complex (Supplementary Figure 3B) showed sparse and weak correlation signals, with noticeably weaker and more fragmented patterns throughout the helices and active site. This suggests that, in the absence of the bioactive ligand, the dynamic coupling between key structural regions is reduced, leading to greater local flexibility, less coherent collective motion, and diminished allosteric coordination. Overall, these results highlight the capacity of Phenol, 2,5-bis(1,1-dimethylethyl)- (Phenol-derived compound) to reinforce dynamic communication and maintain structural cohesion within ESR1, potentially influencing its regulatory role by favoring a more stable and functionally competent conformation compared to the standard complex.

Similarily, Principal component analysis (PCA) was examined for TNF-alpha to evaluate the influence of Gamma-sitosterol binding relative to its standard reference compound. The PCA projection for the TNF-alpha–Gamma-sitosterol complex (Figure 14C) revealed a broader and bifurcated cluster distribution along the first two principal components (PC1 and PC2), accompanied by a distinct color gradient transition from dark blue to yellow that traces the temporal evolution of the simulation over 300 ns. This wider spread and clear gradient progression indicate that Gamma-sitosterol induces multiple metastable states and maintains a dynamic equilibrium, allowing TNF-alpha to sample a more diverse conformational space while retaining structural stability. In contrast, the standard TNF-alpha complex (Supplementary Figure 3C) exhibited a compact and narrowly confined cluster with minimal spread around the centroid and color points remaining tightly grouped. This pattern reflects a stable but conformationally restricted state, with reduced flexibility and limited exploration of alternative structural arrangements. Overall, these observations suggest that Gamma-sitosterol promotes enhanced conformational adaptability in TNF-alpha compared to the standard ligand, potentially contributing to its stabilizing effect on the cytokine functional regions. The Dynamic Cross-Correlation Matrix (DCCM) analysis provides valuable insights into how Gamma-sitosterol binding modulates the collective internal dynamics of TNF-α compared to its standard reference complex. The DCCM plot for the TNF-alpha–Gamma-sitosterol complex (Figure 13D) shows extensive regions of both positive (red) and negative (blue) correlations throughout the matrix, indicating well-coordinated, large-scale residue fluctuations. Notably, the helical regions surrounding the active site display strong correlated motions, suggesting that Gamma-sitosterol binding enhances dynamic communication among these key secondary structure elements. This reinforced coupling likely helps stabilize the ligand-binding pocket while maintaining the overall structural integrity necessary for receptor function. In contrast, the DCCM for the standard TNF-alpha complex (Supplementary Figure 3D) exhibits more localized and fragmented correlation patterns, with isolated pockets of anti-correlated motions but less extensive positive correlations overall. The reduced connectivity between helical segments and the active site indicates that, without Gamma-sitosterol, the dynamic coupling within TNF-alpha is weaker and less synchronized, resulting in greater local flexibility but less coherent domain-level motion. Therefore, these findings reveal a consistent dynamic pattern across both targets, showing that the phytochemical ligands, (Phenol-derived for ESR1 and Gamma-sitosterol for TNF-alpha) promote greater conformational stability, stronger inter-residue correlations, and coordinated structural dynamics than their respective standard inhibitors. This enhanced dynamic behavior may contribute to improved binding affinity and functional modulation, supporting the potential of these compounds as multi-target phytotherapeutic agents capable of influencing key regulatory pathways.

##### 8.5.3.2 RG-RMSD-based free energy landscape

**FIGURE 15:**
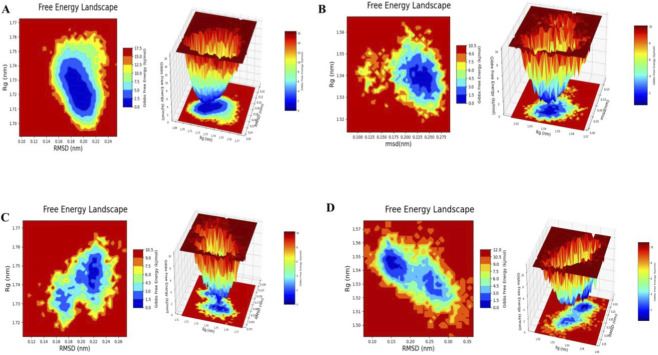
Free energy landscapes (FEL) of protein–ligand complexes projected along RMSD and radius of gyration (Rg). **(A,B)** Phytochemical-bound systems showing dominant low-energy conformational basins indicative of stable binding. **(C)** ESR1 standard ligand complex and **(D)** TNF-α standard ligand complex used for comparison. Color scale represents Gibbs free energy, with blue indicating low-energy stable states and red representing higher-energy conformations.

For the ESR1–Phenol complex, The resulting free energy landscape (Figure 15A) displays a well-defined, narrow low-energy basin located around RMSD ≈0.18–0.22 nm and Rg ≈ 1.73–1.75 nm, indicating a thermodynamically stable and compact conformation. The corresponding Gibbs free energy spans from 0 to ∼17.5 kJ/mol, with the global minimum representing the most stable state sampled throughout the 300 ns simulation. The 3D FEL further reinforces this, showing a steep energy funnel converging around the low-energy region, which reflects minimal structural fluctuation and enhanced stability due to ligand binding. In contrast, the standard ESR1 complex (Figure 15C) presents a broader and more shallow energy distribution with multiple local minima spanning RMSD ≈0.16–0.24 nm and Rg ≈ 1.72–1.77 nm. The associated Gibbs free energy ranges from 0 to ∼10.5 kJ/mol, suggesting greater conformational variability and lower thermodynamic stability than the Phenol-bound system. These differences support the conclusion that Phenol-derived compound binding stabilizes ESR1, both structurally and energetically, by favoring a more defined, low-entropy state.

Similarly, FEL analysis was extended to the TNF-alpha complexes. The TNF-alpha–Gamma-sitosterol complex (Figure 15B) exhibits a compact and sharply defined low-energy basin, centered around RMSD ≈0.20–0.25 nm and Rg ≈ 1.53–1.55 nm, with a Gibbs free energy range of approximately 0–10.5 kJ/mol. This reflects a thermodynamically stable conformation with limited structural drift, implying a rigid and energetically favorable binding mode maintained throughout the simulation. The 3D FEL further illustrates a steep, dominant energy funnel, consistent with a single, stable binding-induced structural state. In contrast, the TNF-alpha–standard complex (Figure 15D) reveals a wider and more distributed energy landscape, characterized by multiple shallow minima and a Gibbs free energy range of 0–∼12.5 kJ/mol. This indicates increased conformational heterogeneity and reduced thermodynamic ordering, suggesting a less efficient stabilization of TNF-alpha by the standard ligand. Comparing these FELs across systems highlights a consistent trend: both Phenol-derived compound and Gamma-sitosterol promote narrow, well-defined energy basins, suggesting that phytochemical binding leads to enhanced conformational stability and restricted fluctuation. Conversely, the standard inhibitors induce broader, more heterogeneous energy landscapes, reflecting higher structural flexibility and lower stability. These thermodynamic observations align well with PCA and DCCM analyses, collectively demonstrating that phytochemical ligands confer greater structural order and functional stability to both ESR1 and TNF-alpha. Crucially, because the Free Energy Landscape (FEL) plots were constructed using the RMSD and Rg trajectories using the Geo_measure 0.9 plugin in PyMOL, the coordinates of these highly stable low-energy basins perfectly correspond to the average RMSD and Rg values computed in our earlier trajectory analysis. This exact alignment serves as a robust cross-validation of the structural parameters, proving the high reproducibility of the simulations and mathematically confirming the consistent stabilizing effects of the phytochemicals. This underscores their potential as effective modulators in targeted therapy.

### 8.6 MM-PBSA and energy decomposition analysis

**FIGURE 16:**
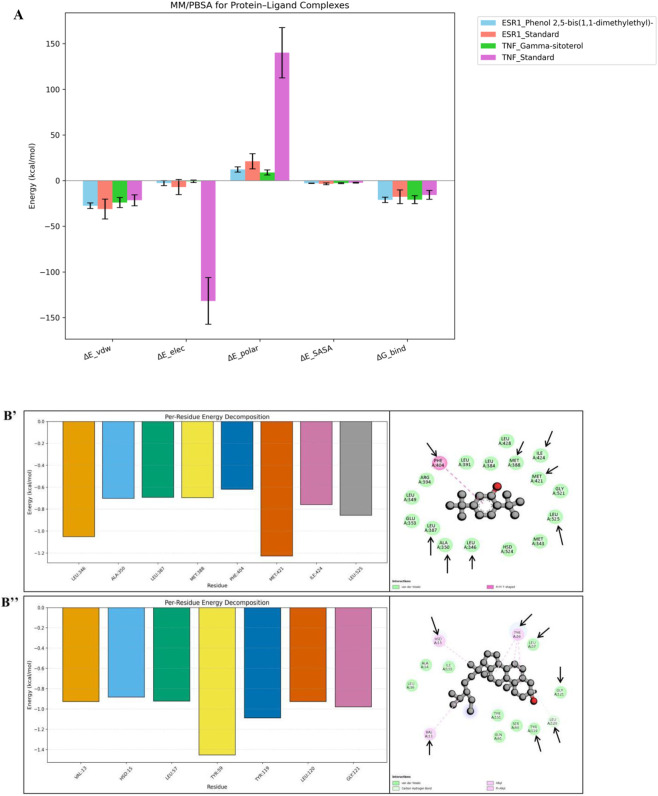
MM/PBSA binding free energy and residue-wise energy decomposition analysis of ESR1 and TNF-alpha protein–ligand complexes. **(A)** Estimated binding free energies for ESR1 and TNF-alpha protein–ligand complexes calculated using MM/PBSA. The bar plot shows the MM/PBSA-derived van der Waals (ΔEvdW), electrostatic (ΔEelec), polar solvation (ΔEpolar), non-polar solvation (ΔESASA), and total binding free energies (ΔGbind) for ESR1 bound to Phenol, 2,5-bis(1,1-dimethylethyl)- (blue) and the standard ligand (red), and TNF-alpha bound to Gamma-sitosterol (green) and the standard ligand (purple). **(B)** The energy decomposition was calculated using the MM-PBSA approach. The results were plotted over 300 ns of the molecular dynamics simulation for each complex. **(B’)** represents the ESR1– Phenol 2,5-bis(1,1-dimethylethyl)- complex, and **(B”)** corresponds to the TNF–Gamma-sitosterol complex. The molecular interactions between the ligand and surrounding residues are shown in the respective panels. It should be noted that the HSD residue refers to histidine (HIS).

A critical factor in molecular interaction is the binding energy, as it encapsulates the overall thermodynamic favorability of a ligand–receptor complex. This metric integrates multiple energetic contributions including van der Waals forces, electrostatics, polar solvation, and non-polar solvation (SASA) to provide a holistic assessment of binding strength and stability. Generally, more negative binding free energy values indicate stronger and more favorable interactions between the ligand and the protein target. To quantitatively evaluate the binding efficiency and stability of the studied phytochemicals, we computed the MM/PBSA binding free energies for four systems, ESR1 with phenol-derived compound, ESR1 with the standard ligand, TNF-alpha with Gamma-sitosterol, and TNF-alpha with its reference standard. These data, summarized in Table 3; Figure 16, with the exact standard deviations for all energy components detailed in the Supplementary Material. The critical insights into the comparative binding profiles and inhibitory potential of the natural compounds relative to known standards in both the ESR1–ligand and TNF-alpha–ligand complexes.

**TABLE 3:** Binding free energies (kcal/mol) of ESR1 bound to Phenol and the standard (native ligand), and TNF-alpha bound to Gamma-sitosterol and the standard (native ligand), as calculated by MM/PBSA analysis over a 300 ns molecular dynamics simulation.

Energies (kcal/mol)	ESR1		TNF-alpha	
	Phenol, 2, 5- bis (1,1-dimethylethyl)-	Standard	Gamma-sitosterol	Standard
Van der Waal energy (kcal/mol)	−27.59 ± 3.03	−31.27 ± 10.87	−24.09 ± 5.57	−21.62 ± 5.95
Electrostatic energy (kcal/mol)	−2.70 ± 2.75	−7.07 ± 8.29	−0.81 ± 1.44	−131.82 ± 25.52
Polar solvation energy (kcal/mol)	12.14 ± 2.79	24.07 ± 8.30	6.78 ± 2.76	140.06 ± 27.49
SASA energy (kcal/mol)	−3.03 ± 0.17	−3.59 ± 1.01	−2.85 ± 0.54	−2.38 ± 0.48
Binding energy (kcal/mol)	−21.18 ± 2.92	−17.86 ± 7.50	−20.97 ± 4.37	−15.76 ± 4.86

In the ESR1 system, Phenol-derived phytochemical demonstrated a slightly more favorable binding free energy (−21.18 ± 2.92 kcal/mol) compared to the standard ligand (−17.86 ± 7.50 kcal/mol). Although the standard exhibited stronger van der Waals (−31.27 kcal/mol vs. −27.59 kcal/mol) and electrostatic interactions (−7.07 kcal/mol vs. −2.70 kcal/mol), its significantly higher polar solvation penalty (+24.07 kcal/mol) diminished its overall binding efficiency. In contrast, the phenol derivative incurred a substantially lower polar solvation energy (+12.14 kcal/mol), which compensated for its comparatively weaker direct interactions. Both ligands contributed favorably through non-polar solvation energy, with the standard showing slightly greater hydrophobic burial (−3.59 vs. −3.03 kcal/mol).

For the TNF-alpha complexes, Gamma-sitosterol exhibited a markedly favorable binding free energy (−20.97 ± 4.37 kcal/mol), whereas the standard ligand displayed a highly unfavorable total energy (−15.76 ± 4.86 kcal/mol). This striking difference primarily stems from the extreme polar solvation penalty associated with the standard (+140.06 kcal/mol), which outweighed its strong electrostatic interactions (−131.82 kcal/mol). In contrast, Gamma-sitosterol maintained a more balanced energetic profile with moderate van der Waals (−24.09 kcal/mol), minimal electrostatic interactions (−0.81 kcal/mol), and a much lower desolvation energy (+6.78 kcal/mol), supporting a more thermodynamically stable and biologically relevant interaction. These results collectively underscore the superior binding efficiency of the natural compounds over their respective standards. Notably, the balance between favorable interactions (electrostatic and van der Waals) and solvation penalties emerged as a key determinant of binding affinity. The energetic profiles of Phenol 2,5-bis(1,1-dimethylethyl)- and Gamma-sitosterol aligned well with their docking predictions, reinforcing their potential as effective inhibitors of ESR1 and TNF-alpha, respectively. Thus, these findings support the therapeutic promise of *Arisaema Jacquemonti-*derived phytochemicals as multi-target modulators for atherosclerosis and inflammation related diseases.

To precisely evaluate the binding affinities of protein–ligand system, Molecular Mechanics Poisson–Boltzmann Surface Area (MM-PBSA) calculations were performed, incorporating per-residue energy decomposition. Unlike conventional molecular docking, which provides static interaction predictions, simulation-based methods such as MM-PBSA offer dynamic and thermodynamically relevant insights into ligand binding Per-residue energy decomposition was performed over the last 300 ns of the molecular dynamics (MD) simulation to pinpoint residues significantly influencing ligand recognition and binding stability at the active site. In the ESR1–Phenol complex, the decomposition profile revealed a prominent cluster of hydrophobic residues facilitating ligand stabilization predominantly via van der Waals and non-polar interactions. Among these, MET421 emerged as the primary contributor (−1.22 kcal/mol), acting as a central anchoring residue. The persistent hydrophobic interactions between its side chain and the bulky tert-butyl groups of the ligand likely support optimal positioning within the receptor binding cavity. Additional key residues LEU346 (−1.05 kcal/mol), LEU525 (−0.86 kcal/mol), and ILE424 (−0.76 kcal/mol) encircle the ligand to form a hydrophobic pocket that reinforces conformational stability. MET388 and ALA350 (each contributing −0.70 kcal/mol) further enhance this hydrophobic environment. These findings are in close concordance with molecular docking predictions, validating their structural and functional importance in ligand anchoring and receptor engagement (Figure 16B’).

In the case of the TNF–Gamma-sitosterol complex, per-residue decomposition analysis (Figure 16B”) identified TYR59 as the most significant contributor (−1.45 kcal/mol). Its interaction is likely mediated via π-alkyl or hydrophobic contacts with the sterol ring, highlighting it as a critical interaction hotspot within the TNF binding cleft. Additional residues, including TYR119 (−1.09 kcal/mol), GLY121 (−0.98 kcal/mol), and LEU120 (−0.92 kcal/mol), also displayed notable contributions, predominantly through backbone interactions and hydrophobic stabilization. Other significant residues, such as LEU57 (−0.91 kcal/mol), HIS15 (−0.83 kcal/mol), and VAL13 (−0.89 kcal/mol), further reinforced the nonpolar environment necessary for effective ligand binding. These interaction patterns are consistent with prior docking studies, which identified the same residues as central to ligand stabilization and specificity. In conclusion, the MM-PBSA-based energy decomposition analysis supports the thermodynamic favorability and structural stability of the ESR1–Phenol and TNF-Gamma-sitosterol complexes over the course of the simulation.

Collectively, the integrated outcomes from molecular docking, interaction profiling, MD simulations, MM-PBSA free energy calculations and decomposition analysis demonstrate a high degree of concordance, providing deep mechanistic insight and reinforcing the robustness and reliability of the findings. Notably, the key interacting residues identified through molecular interaction profiling were critically cross-validated by per-residue energy decomposition, while the binding affinities obtained from docking closely mirrored the MM-PBSA results. Furthermore, the MD simulations confirmed the dynamic stability of both ESR1-Phenol and TNF–Gamma-sitosterol complexes throughout the simulation period, and these findings are heavily supported by existing experimental literature on the structural vulnerabilities of these targets (e.g., the ESR1 Helix-12 selectivity switch and the TNF-alpha monomer interface). This convergence across multiple computational approaches provides strong, mutually supportive evidence that the selected bioactive compounds from *Arisaema Jacquemontii Blume* not only bind effectively but also maintain stable, energetically favorable interactions with ESR1 and TNF-alpha. These consistent findings underscore their potential as reliable, multi-target natural inhibitors for therapeutic applications, particularly in inflammation-mediated cardiovascular disorders.

## 9 DFT studies

### 9.1 Geometry optimization

The computational examination of chemical compounds often relies on Density Functional Theory (DFT) due to its strength and reliability. Exploring the molecular structure and chemical reactivity of bioactive molecules is essential for understanding their potential biological roles and mechanisms of action. In this study, the molecular geometries of the two selected phytochemicals were optimized to their lowest energy configurations using the specified DFT basis set. This procedure produced structurally stable conformations with reduced steric clashes and optimized bound angles, providing valuable insights into their chemical properties and potential biological activity.

**FIGURE 17:**
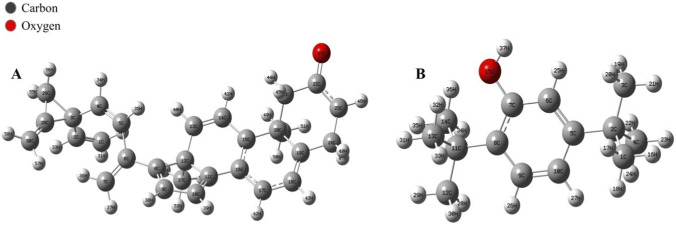
The optimized geometries of **(A)** Gamma-sitosterol and **(B)** Phenol-derived compound at B3LYP/6-31G(d).

The DFT optimized molecular structures of Gamma-sitosterol and Phenol-derived compound (Figure 17) exhibit distinct structural characteristics that contribute to their chemical stability and potential biological interactions. In Gamma-sitosterol, the hydroxyl group at carbon 3 (C-3) serves as the primary polar functional group and can participate in hydrogen-bonding interactions as both a donor and an acceptor. The molecule possesses a rigid, non-planar tetracyclic sterol core and a flexible aliphatic side chain, enabling favorable accommodation within hydrophobic binding pockets and promoting van der Waals and hydrophobic interactions. In contrast, Phenol-derived compound (Figure 17B) adopts a planar aromatic conformation due to its benzene ring, substituted at positions 2 and 5 with bulky tert-butyl groups, and features a hydroxyl group at carbon 7 (7C), linked to oxygen atom 15O. The planarity of the aromatic ring facilitates π–alkyl interactions with adjacent non-polar groups, while the phenolic–OH group enables directional hydrogen bonding. The optimized geometries of both compounds display ideal bond angles and minimal steric hindrance, enhancing their ability to interact with biological macromolecules. Their complementary amphiphilic profiles, combining hydrophilic and hydrophobic regions with conformational stability, support their potential roles as anti-inflammatory and cardiovascular protective agents.

### 9.2 Frontier molecular orbital (FMO) and molecular electrostatic potential

**FIGURE 18:**
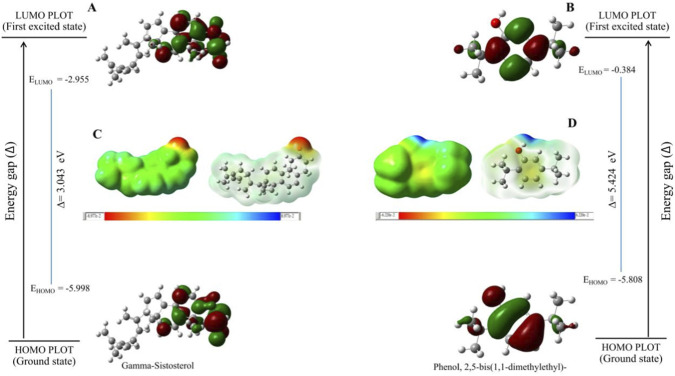
Frontier molecular orbitals and molecular electrostatic potential (MEP) surfaces of Gamma-sitosterol and Phenol, 2,5-bis(1,1-dimethylethyl)- were computed using DFT. **(A,B)** depict the Highest Occupied Molecular Orbital (HOMO) and Lowest Unoccupied Molecular Orbital (LUMO) for Gamma-sitosterol and Phenol, 2,5-bis(1,1-dimethylethyl)-, respectively, highlighting the spatial localization of electron density in their ground and excited states. **(C,D)** present the corresponding MEP maps, illustrating regions of high and low electron density for each compound. The HOMO–LUMO energy gap (ΔE) is reported for both molecules, offering insights into their chemical reactivity and kinetic stability. The electrostatic potential is color-coded across the molecular surface, with the gradient increasing in the order: red < orange < yellow < green < blue.

Frontier molecular orbital (FMO) analysis provides insight into molecular electronic structure, highlighting electron-donating (HOMO) and electron-accepting (LUMO) properties (Figures 18A,B). The energy difference between these orbitals, the HOMO-LUMO gap, directly indicates chemical reactivity and kinetic stability, where a smaller gap indicates increased electronic reactivity and a larger gap implying greater electronic stability (Kamel et al., 2025). In this study, Gamma-sitosterol exhibited a relatively smaller energy gap of 3.043 eV, suggesting it is comparatively softer and more reactive. Biologically, this chemical softness perfectly aligns with our MD simulation findings, where Gamma-sitosterol demonstrated the dynamic conformational adaptability required to stably anchor within the complex TNF-alpha interface. Conversely, Phenol-derived compound yields a significantly larger energy gap of 5.424 eV, indicating greater electronic stability and a reduced tendency for electron acceptance. This high kinetic stability correlates directly with its rigid, sustained binding profile within the ESR1 hydrophobic pocket, ensuring a durable blockade of the receptor without undergoing rapid degradation.

Molecular Electrostatic Potential (MEP) surface analysis is an advanced computational approach employed to depict the spatial distribution of electrostatic charge across a molecule. Integrating these MEP maps with our molecular docking data provides a clear quantum-level rationale for the observed mechanisms of action. For Gamma-sitosterol, a distinct red region localized around the carbonyl oxygen at the 25O position signifies an electron-rich, highly electronegative site. This specific electronegative pocket facilitates the critical polar interactions and hydrogen bonding required to disrupt the TNF-alpha pro-inflammatory signaling cascade, directly supporting the strong −7.0 kcal/mol binding affinity observed in docking. Conversely, the MEP map of the Phenol-derived compound exhibited a pronounced blue region centered on the hydroxyl hydrogen, reflecting its role as a strong hydrogen bond donor and an electrophilic interaction site. This quantum descriptor directly explains the compound’s mechanism of action: the highly positive (blue) hydroxyl group is electronically primed to form the crucial 2.6 Å hydrogen bond with the Leu346 residue in the ESR1 active site, as observed in our docking models. Furthermore, the surrounding aromatic ring displayed a neutral green surface, which facilitates the essential van der Waals and π–alkyl interactions with surrounding non-polar residues like Ala350 and Leu525. By utilizing this specific electronic distribution to anchor to His524 and Leu525 the critical “selectivity switch” of ESR1, the compound effectively alters the orientation of Helix 12, blocking co-activator recruitment and turning off pro-inflammatory gene expression (Figures 18C,D).

In summary, the MEP and FMO analyses demonstrate that the specific electronic configurations of these compounds are not merely descriptive, but are the fundamental biophysical drivers that enable their precise recognition, stable binding, and subsequent biological modulation of the ESR1 and TNF-alpha targets.

### 9.3 Chemical reactivity descriptors

The quantum chemical descriptors derived from DFT calculations offers valuable insights into the electronic configuration and reactivity of Gamma-sitosterol and Phenol, 2,5-bis(1,1-dimethylethyl)- (Table 4). For Gamma-sitosterol, the HOMO and LUMO energy levels are −5.998 eV and −2.955 eV, respectively, resulting in a moderate energy gap of 3.043 eV. This suggests a balanced profile between kinetic stability and chemical reactivity. The ionization potential (5.998 eV) and electron affinity (2.955 eV) indicate the molecule capacity to engage in both electron donation and acceptance. The computed electronegativity (4.476 eV) and chemical potential (−4.476 eV) point to a strong tendency to attract electrons, while its relatively low chemical hardness (1.521 eV) and higher softness value (0.328 eV) classify it as a chemically soft species, favouring reactivity. A notably high dipole moment of 8.346 Debye further underscores the compound’s polar character and its potential for interactions in aqueous or polar biological environments. The maximum electronic charge that the molecule can accept (ΔN_max_) is 1.471, supporting its ability to participate in charge transfer processes.Additionally, the CH-bending and stretching vibrational frequencies, observed at 17.91 cm^−1^ and 3297.41 cm^−1^, respectively, provide further evidence of the molecule structural integrity and correlate with characteristic peaks in the IR spectrum Supplementary Figure 4.

**TABLE 4:** Quantum parameters for Gamma-Sitosterol and Phenol-derived compound by the B3LYP/6-31G(d) method.

Quantum parameter	Gamma-sitosterol	Phenol -derived compound
E_HOMO_/eV	−5.998	−5.808
E_LOMO_/eV	−2.955	−0.384
Energy/eV	3.043	5.424
Ionization potential, IP (eV)	5.998	5.808
Electron affinity, EA (eV)	2.955	0.384
Electronegativity, χ (eV)	4.476	3.096
Chemical potential (μ)	−4.476	−3.096
Chemical hardness (ƞ)/eV	1.521	2.712
Chemical softness (s)/eV	0.328	0.184
Dipole moment/Debye	8.346	2.478
Maximum charge transfer	1.471	0.570
CH-bending frequency	17.91	36.00
CH-stretching frequency	3297.41	3746.40

In contrast, Phenol-derived compound displays HOMO and LUMO energy level of −5.808 eV and −0.384 eV, respectively, yielding a significantly wider energy gap of 5.424 eV, indicative of greater kinetic stability and lower reactivity. The ionization potential (5.808 eV) and electron affinity (0.384 eV) reflect its electronic disposition in redox systems. The electronegativity (3.096 eV) and chemical potential (−3.096 eV) suggest moderate electron-withdrawing capability. Its higher hardness (2.712 eV) and lower softness (0.184 eV) classify it as a hard molecule, typically associated with chemical inertness. The compound’s dipole moment of 2.478 Debye reflects lower polarity, which could limit its solubility and interaction with polar environments. Its vibrational features, including CH-bending and CH-stretching frequencies at 36.00 cm^−1^ and 3746.40 cm^−1^, respectively, are consistent with its rigid structure and are supported by IR spectral data (Supplementary Figure 5). The maximum charge transfer (ΔN_max_), calculated at 0.570, further confirms its limited capacity for electron redistribution compared to softer, more reactive molecules. Overall, these quantum chemical parameters highlight the contrasting electronic and physicochemical profiles of the two compounds. Gamma-sitosterol, with its greater polar character, chemical softness, charge-transfer capacity, and flexible electron density, demonstrates features conducive to biological reactivity and interaction, supporting its potential as a pharmacologically active molecule. Conversely, Phenol-derived compound shows greater electronic stability but lower reactivity, suggesting a more inert role within biological systems. These distinct quantum-level profiles help explain their differing binding affinities and interaction behaviors, as further supported by molecular docking and molecular dynamics simulation results. Overall, this comparative DFT analysis provides crucial quantum-level validation for the studied phytochemicals, demonstrating how their distinct electronic properties fundamentally shape their anticipated therapeutic potential. By correlating specific chemical descriptors to distinct target microenvironments, these findings highlight the unique contributions of both compounds to the predicted cardioprotective effects of *Arisaema Jacquemontii Blume*. As a next step, we will conduct detailed *in silico* bioactivity analyses to further substantiate and refine these computational predictions.

## 10 *In silico* prediction of bioactive compound reactivity and metabolism

**FIGURE 19:**
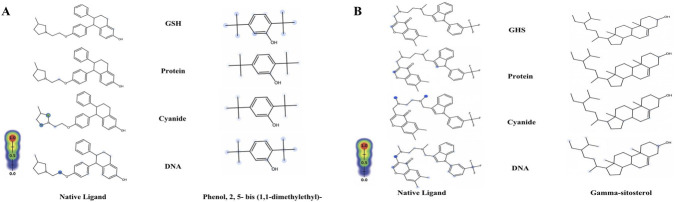
XenoSite-predicted reactive sites of standard (native ligands) and phytochemicals. **(A)** Predicted interaction hotspots of the standard and the phenol-derived compound, showing potential reactivity toward GSH, proteins, cyanide, and DNA. **(B)** Predicted reactive sites of the standard and Gamma-sitosterol highlighting possible GSH, protein, cyanide, and DNA interactions. Light-blue regions indicate a low probability of forming reactive intermediates.

The bioactive compounds, along with their standard references, were evaluated using the reactivity module in XenoSite as shown in Figures 19A,B. According to *in silico* predictions and existing literature, potential metabolites and reactive intermediates were identified. Using the XenoSite reactivity prediction model, electrophilic hotspots on the standard (native ligand) and bioactive compounds were assessed for their theoretical susceptibility to interactions with GSH, proteins, cyanide, and DNA. The predicted reactivity at each atomic site was visualized using a gradient color scale, where red signified a high likelihood of reactive intermediate formation, and white denoted minimal or no reactivity at the site, whereas the faint blue color represents a lower probability of forming reactive intermediates. The cyanide reactivity model identified the pyrrolidine ring with methyl substituents in the standard as the most reactive site, as evident from the dark blue region. This suggests a high probability of oxidative processes leading to the formation of reactive electrophilic intermediates, which may readily interact with cyanide or other nucleophilic species. Additionally, the DNA reactivity model highlighted a strong preference for bioactivation at the N-linked ethoxy functional group, indicating a high predicted likelihood of DNA adduct formation and a potential genotoxic risk associated with the ESR1-standard. In contrast, Phenol-derived compound exhibited no highly reactive dark blue zones, signifying a significantly lower computational probability of metabolic bioactivation and reduced susceptibility to reactive metabolite formation. The steric hindrance provided by the bulky tert-butyl (-C(CH3)3) groups at the second and fifth positions is predicted to act as a protective shield around the phenol moiety, minimizing its susceptibility to oxidative degradation and enhancing its overall chemical stability. This structural feature reduces the potential for toxic metabolite generation, suggesting a highly favorable safety profile for drug development, especially when compared to the ESR1-native-ligand. Similarly, the reactivity analysis of Gamma-sitosterol and its corresponding standard revealed distinct patterns of reactive site distribution. The standard (native ligand) exhibited dark blue regions, indicating highly reactive sites primarily located at the ether group, amide functional groups, and electron-rich aromatic rings. These regions suggest strong interactions with biomolecules via hydrogen bonding, nucleophilic attacks, or electrophilic reactions, making the standard (native ligand) prone to metabolic reactivity with cyanide, proteins, and DNA. Conversely, Gamma-sitosterol lacked any dark blue zones, indicating a lack of significant reactivity under the given predictive conditions. Its structure, consisting of a steroidal backbone with a hydroxyl functional group, demonstrated high theoretical chemical stability, minimizing the risk of bioactivation or toxic metabolite formation (Figure 19B). Overall, both Phenol 2,5-bis(1,1-dimethylethyl)- and Gamma-sitosterol exhibit strong computational metabolic stability and reduced reactivity. Their lower susceptibility to oxidative bioactivation suggests a decreased likelihood of forming harmful reactive intermediates, which may contribute to enhanced bioavailability, metabolic resilience, and improved safety profiles in biological systems. These properties underscore their potential as promising predictive therapeutic agents for cardiovascular disease intervention, particularly in the prevention and management of atherosclerosis and other inflammation-related diseases.

The SMARTCyp analysis predicts that both the standard and Phenol-derived compound share C.6 as a common metabolic hotspot, indicating that this position is a key site for CYP3A4-mediated oxidation. However, while the ESR1 standard (native ligand) exhibits moderate metabolic susceptibility (C.6 score ∼34.2), Phenol-derived compound demonstrates a higher metabolic energy (∼77.2) and lower solvent accessibility (2DSASA ∼19.8), suggesting it may be less prone to rapid degradation. The increased energy requirement for oxidation indicates that the bioactive compound is metabolically more stable, theoretically leading to a longer half-life and sustained activity. Furthermore, its lower accessibility to CYP3A4 enzymes may contribute to improved bioavailability compared to the native ligand. This suggests that Phenol-derived phytochemical could serve as a metabolically favorable candidate for targeting ESR1, offering enhanced therapeutic potential with reduced metabolic clearance. Similarly, the SMARTCyp analysis for the TNF standard (native ligand) and Gamma-sitosterol reveals distinct metabolic susceptibilities. The native-ligand shows major CYP3A4-mediated oxidation hotspots at C.16, C.20, and C.14, with metabolic scores ranging from 33.8 to 35.1, and a high 2DSASA value (∼53.3 for C.16 and C.20), indicating significant solvent exposure and increased likelihood of metabolic degradation. Conversely, Gamma-sitosterol exhibits metabolic hotspots at C.23, C.24, and C.18, with significantly higher metabolic energy (62.2–66.4), suggesting that it is more metabolically stable than the TNF-native-ligand. The lower 2DSASA values further indicate reduced accessibility to CYP3A4, potentially leading to slower metabolic clearance and prolonged systemic presence. Overall, the bioactive compounds (Phenol 2,5-bis(1,1-dimethylethyl)- and gamma-sitosterol) demonstrate substantial predicted metabolic resilience. Nevertheless, the phytochemicals' inherent increased stability may contribute to better pharmacokinetic properties, enhanced bioavailability, and prolonged biological activity. Their reduced susceptibility to CYP3A4-mediated metabolism suggests slower clearance rates, which could lead to sustained therapeutic effects (Supplementary Figure 6).

These metabolic and reactivity findings align well with the quantum-level validation provided by the DFT analysis. The DFT results demonstrated that Gamma-sitosterol possesses favorable chemical softness, strong polarity, and charge-transfer potential, consistent with its predicted chemical stability and controlled reactivity under metabolic conditions. Meanwhile, Phenol-derived compound was shown to be electronically stable and sterically protected, supporting its observed computational resistance to oxidative bioactivation and limited formation of harmful intermediates. Altogether, the combined predictive evidence from XenoSite, SMARTCyp, and DFT calculations suggests that Phenol-derived compound and Gamma-sitosterol exhibit strong metabolic resilience, reduced reactivity, and enhanced chemical stability. These properties suggest improved bioavailability, slower metabolic clearance, and a lower risk of generating toxic metabolites, strengthening their promise as safe and effective candidates for cardiovascular intervention that require future *in vitro* validation. Their integrated reactivity and quantum-level profiles highlight the value of combining advanced *in silico* tools to de-risk early-stage phytochemical drug discovery.

## 11 Computational assessment of bioactivity for bioactive compounds

Table 5 summarizes the predicted activities relevant to atherosclerosis, providing preliminary insights into their possible therapeutic roles. However, these predictions should be interpreted cautiously, as they do not confirm experimental efficacy.

**TABLE 5:** Predicted biological activity scores of the selected bioactive compounds using the PASS Online server.

SL no.	Bioactive names			Bioactivity
1	Phenol-derived compound	Pa	0.828	Beta-adrenergic receptor kinase inhibitor
		Pi	0.010	
		Pa	0.828	G-protein-coupled receptor kinase inhibitor
		Pi	0.010	
		Pa	0.799	JAK2 expression inhibitor
		Pi	0.008	
		Pa	0.792	Lipoprotein lipase inhibitor
		Pi	0.008	
		Pa	0.772	NADPH peroxidase inhibitor
		Pi	0.016	
		Pa	0.770	Antiinflammatory
		Pi	0.009	
2	Gamma-sitosterol	Pa	0.960	Antihypercholesterolemic
		Pi	0.002	
		Pa	0.957	Cholesterol antagonist
		Pi	0.001	
		Pa	0.924	Hypolipemic
		Pi	0.004	
		Pa	0.886	Oxidoreductase inhibitor
		Pi	0.003	
		Pa	0.831	Chemopreventive
		Pi	0.003	
		Pa	0.745	HMOX1 expression enhancer
		Pi	0.005	

For Phenol 2,5-bis(1,1-dimethylethyl)-, the PASS analysis predicted several cardiovascular-related activities, including beta-adrenergic receptor kinase inhibition, G-protein-coupled receptor kinase (GPRK) inhibition, JAK2 expression inhibition, lipoprotein lipase inhibition, NADPH oxidase inhibition, and anti-inflammatory activity. The predicted Pa values ranged from 0.770 to 0.828, with corresponding Pi values between 0.008 and 0.016, indicating a reasonable probability of activity. These predicted activities suggest a potential role in modulating cardiovascular processes such as inflammation, oxidative stress, and lipid metabolism. Beta-adrenergic receptor kinase and GPRK inhibition have been associated with improved cardiac function and restoration of beta-adrenergic signaling (Cannavo et al., 2013; Miao et al., 2017), while JAK2 signaling is known to contribute to inflammation and vascular remodeling in cardiovascular disease.

Similarly, Gamma-sitosterol exhibited predicted activities associated with cholesterol regulation, including antihypercholesterolemic, cholesterol antagonist, hypolipidemic, oxidoreductase inhibition, chemopreventive effects, and HMOX1 expression enhancement. The Pa values for these activities ranged from 0.745 to 0.960, with low Pi values (0.001–0.005), indicating a high likelihood of activity. These predictions are consistent with previously reported roles of phytosterols in modulating lipid metabolism and oxidative stress. The antihypercholesterolemic and cholesterol antagonist properties of Gamma-sitosterol may be attributed to its structural similarity to beta-sitosterol, a well-characterized phytosterol known to reduce intestinal cholesterol absorption (El Omari et al., 2024; Khan et al., 2022). In addition, the predicted oxidoreductase inhibitory and HMOX1-inducing activities suggest a potential role in reducing oxidative stress and supporting vascular homeostasis. This evidence not only enhances the credibility of the PASS Online bioactivity prediction model but also highlights its utility in identifying compounds with comparable structures, such as Gamma-sitosterol. These results emphasize the importance of computational tools in accelerating drug discovery by identifying bioactive compounds and predicting their effects before *in vitro* validation, streamlining development across various therapies.

## 12 ADMT evaluation and toxicity analysis

The *in silico* ADMET analysis evaluated the pharmacokinetic profiles of the Phenol-derived compound and Gamma-sitosterol. While the native co-crystallized ligands were included in the evaluation as structural references, it is important to note that these synthetic inhibitors are not typically optimized for oral administration; therefore, the predicted pharmacokinetic profiles of the phytochemicals highlight their distinct suitability for future oral drug development rather than a direct therapeutic superiority (Table 6). Both Phenol-derived compound (predicted 91.359%) and Gamma-sitosterol (predicted 94.464%) exhibit high intestinal absorption scores, suggesting favorable theoretical bioavailability. In addition, moderate Caco-2 permeability values indicate the potential for effective intestinal uptake. Gamma-sitosterol demonstrated higher predicted blood-brain barrier (BBB) permeability (0.781 log BB) than the Phenol-derived compound (0.469 log BB), which may indicate a greater capacity for central distribution. Notably, both phytochemicals were predicted to be non-substrates of P-glycoprotein (P-gp), in contrast to the native ligands, which may influence their cellular retention and transport behavior. In terms of metabolism, the native ligands showed broader interactions with cytochrome P450 (CYP) enzymes, which is expected for synthetic experimental compounds. In contrast, predictive models suggest Phenol 2,5-bis(1,1-dimethylethyl)- exhibited selective inhibition of CYP1A2 and Gamma-sitosterol showed no predicted CYP inhibition, theoretically reducing metabolic complications. The predicted clearance values for Phenol-derived compound (0.753 log ml/min/kg) and Gamma-sitosterol (0.628 log ml/min/kg) indicate moderate elimination profiles, which may support sustained systemic exposure. To evaluate the potential toxicity profiles of the selected bioactive compounds, the OSIRIS Property Explorer was employed to predict toxicity-related molecular properties (Table 7). As these toxicity profiles are purely computational, they must be interpreted cautiously. This analysis provides preliminary insights into drug-likeness and safety based on structural features. The toxicity predictions are color-coded, where “green” indicates no predicted toxicity, “yellow” suggests low toxicity risk, and “orange” and “red” indicate increasing levels of concern. Both bioactive compounds, Phenol-derived compound and Gamma-sitosterol, were predicted to be free from mutagenic, tumorigenic, irritant, and reproductive toxicity risks, suggesting a highly favorable theoretical safety profile at the computational level. In contrast, the native ligands showed predicted toxicity risks, including reproductive and mutagenic effects. The selected phytochemicals also exhibited favorable total polar surface area (TPSA) values (Phenol-derived compound: 20.23 Å^2^; Gamma-sitosterol: 20.23 Å^2^), which may support membrane permeability and absorption. A difference in predicted solubility was observed, with Gamma-sitosterol showing lower solubility (−6.67 log mol/L) compared to the Phenol-derived compound (−3.64 log mol/L), which may influence formulation and bioavailability considerations.

**TABLE 6:** Predicted ADMET properties of the selected bioactive compounds using the pkCSM server.

Properties		Properties		Properties	
		ESR1-native-ligand	Phenol-derived compound	Gamma-sitosterol	TNF-native-ligand
Absorption	Water solubility (log mol/L)	−5.475	−4.711	−6.773	−4.61
	Caco2 permeability ((log papp in 10–6 cm/s)	0.908	1.582	1.201	0.984
	Intestinal absorption (human) (%)	95.559	91.359	94.464	88.949
	P-glycoprotein substrate	Yes	No	No	Yes
	P-glycoprotein I inhibitor	Yes	No	Yes	Yes
	P-glycoprotein II inhibitor	Yes	No	Yes	Yes
Distribution	Fraction unbound (human) numeric (fu)	0.082	0.009	0	0.151
	VDss (human) (log L/kg)	−0.291	0.735	0.193	0.418
	BBB permeability (log BB)	−0.29	0.469	0.781	0.476
	CNS permeability (log PS)	−1.464	−1.052	−1.705	−0.951
Metabolism	CYP3A4 substrate/inhibitor	Yes/No	Yes/No	Yes/No	Yes/Yes
	CYP1A2 inhibitor	No	Yes	No	Yes
	CYP2D6 substrate/inhibitor	Yes/No	No/No	No/No	Yes/Yes
	CYP2C9 inhibitior	No	No	No	No
Excreation	Total clearance (log ml/min/kg)	0.651	0.753	0.628	0.765
	Renal OCT2 substrate	No	No	No	No
Toxicity	AMES toxicity	Yes	No	No	Yes
	Max. Tolerated dose (human) (log mg/kg/day)	0.14	0.537	−0.621	0.534
	Oral rat acute toxicity (LD50) (mol/kg)	2.347	2.137	2.552	1.899
	Oral rat chronic toxicity (LOAEL) (log mg/kg_bw/day)	1.34	1.44	0.855	0.737
	Hepatotoxicity	Yes	No	No	Yes
	T.Pyriformis toxicity (log ug/L)	0.287	1.493	0.43	0.285

**TABLE 7:** Predicted toxicity profiles of the selected bioactive compounds using the OSIRIS Property Explorer. Green indicates no predicted toxicity, yellow denotes mild toxicity, while increasing levels of predicted toxicity are represented by orange and red, respectively.

Compounds	Mutagenic	Tumorigenic	Irritant	Reproductive effects	TPSA	Solubility
ESR1-native-ligand	Green	Green	Green	Red	32.7	−5.08
Phenol-derived compound	Green	Green	Green	Green	20.23	−3.64
Gamma-sitosterol	Green	Green	Green	Green	20.23	−6.67
TNF-native-ligand	Red	Green	Green	Orange	37.71	−7.93

To evaluate the potential toxicity profiles of the selected bioactive compounds, the OSIRIS Property Explorer was employed to predict toxicity-related molecular properties (Table 7). As these toxicity profiles are purely computational, they must be interpreted cautiously. This analysis provides preliminary insights into drug-likeness and safety based on structural features. The toxicity predictions are color-coded, where “green” indicates no predicted toxicity, “yellow” suggests low toxicity risk, and “orange” and “red” indicate increasing levels of concern. Both bioactive compounds, Phenol-derived compound and Gamma-sitosterol, were predicted to be free from mutagenic, tumorigenic, irritant, and reproductive toxicity risks, suggesting a highly favorable theoretical safety profile at the computational level. In contrast, the native ligands showed predicted toxicity risks, including reproductive and mutagenic effects. The selected phytochemicals also exhibited favorable total polar surface area (TPSA) values (Phenol-derived compound: 20.23 Å^2^; Gamma-sitosterol: 20.23 Å^2^), which may support membrane permeability and absorption. A difference in predicted solubility was observed, with Gamma-sitosterol showing lower solubility (−6.67 log mol/L) compared to the Phenol-derived compound (−3.64 log mol/L), which may influence formulation and bioavailability considerations (Table 7).

Overall, the predictive ADMET and OSIRIS-based toxicity profiles suggest that the selected bioactive compounds possess drug-like characteristics, including favorable theoretical absorption, metabolic stability, and safety margins. These computational findings suggest they are viable candidates that warrant rigorous experimental *in vitro* and *in vivo* pharmacokinetic and toxicological evaluation to confirm their safety and efficacy in biological systems.

## 13 Translational implications and limitations of the study

Our integrative findings expand upon previous pharmacological studies of *A. Jacquemontii Blume*. While earlier investigations primarily reported its general antioxidant and anti-inflammatory properties, the present study provides a mechanistic framework suggesting TNF-alpha and ESR1 as key regulatory targets potentially associated with its cardioprotective effects. From a translational perspective, modulation of TNF-alpha may help attenuate inflammatory processes involved in plaque progression, while ESR1-associated pathways are linked to endothelial function and lipid metabolism, both of which are relevant to atherosclerosis pathophysiology.

The rationale for employing an extensive computational framework including network pharmacology, transcriptomic analysis, molecular docking, molecular dynamics simulations, MM/PBSA, and DFT calculations was to systematically prioritize and evaluate phytochemical target interactions at structural, dynamic, and electronic levels. By rigorously cross-validating our findings across these multiple computational approaches and existing experimental literature, we provide deep mechanistic insight and ensure the robustness and reliability of our predictions before experimental investigation. Recent studies have highlighted that integrating molecular simulations can improve the predictive assessment of protein-ligand interactions and facilitate computationally guided therapeutic discovery (Singh et al., 2025; 2022). By applying these complementary methodologies, the present study provides a computational framework that may support future targeted *in vitro* and *in vivo* validation studies of *A. Jacquemontii Blume* in cardiovascular disease.

However, these findings remain purely computational and hypothesis-generating. First, the study relies on a single transcriptomic dataset (GSE236251), which may limit broad generalizability, although our stringent filtering ensures high biological relevance. Furthermore, a notable limitation is our reliance on purely *In silico* models (e.g., pkCSM, OSIRIS, XenoSite, and SMARTCyp) for ADMET, bioactivity, and metabolic profiling. These algorithms cannot fully capture the extraordinary complexity of living biological systems, including physiological barriers, individual metabolic variations, gut microbiome interactions, or unpredicted off-target toxicities. Consequently, the computational pharmacokinetic, metabolic stability, and safety profiles presented herein serve strictly as predictive guidelines rather than definitive proof of clinical safety, metabolic behavior, or absolute target specificity. Although Gamma-Sitosterol and the phenol-derived compound demonstrated favorable computational profiles, including predicted binding stability, pharmacokinetic properties, and target interactions, these observations require further experimental confirmation. Collectively, the present study provides a systems-level computational foundation for guiding future experimental research on plant-derived multi-target therapeutics for atherosclerosis.

## 14 Conclusion

This study establishes a rigorous, multi-tiered computational framework to investigate the cardioprotective potential of bioactive compounds derived from Arisaema Jacquemontii Blume. Through an integrated pipeline encompassing network pharmacology, molecular docking, molecular dynamics (MD) simulations, MM/PBSA thermodynamic assessments, and Density Functional Theory (DFT) analyses, Gamma-sitosterol and Phenol, 2,5-bis(1,1-dimethylethyl)- were identified as highly promising multi-target modulators. Specifically, these phytocompounds demonstrated robust binding affinities, dynamic structural stabilities, and favorable electronic reactivity toward TNF-alpha and ESR1, highlighting their potential to regulate crucial pathways in atherosclerosis, including vascular inflammation and lipid metabolism. While this study provides comprehensive and statistically cross-validated *in silico* evidence, these computational, hypothesis-generating predictions must be interpreted with appropriate scientific caution. To bridge the gap between computational prediction and clinical application, we propose a systematic experimental validation strategy. First, direct biophysical assays, such as Surface Plasmon Resonance (SPR) or Isothermal Titration Calorimetry (ITC), should be employed to empirically confirm the predicted binding affinities of Gamma-sitosterol and the phenol-derived compound. Second, *in vitro* cell-based models, particularly human umbilical vein endothelial cells (HUVECs) or RAW 264.7 macrophage, are required to evaluate their functional ability to inhibit TNF-alpha-induced inflammatory cascades and modulate ESR1-mediated lipid metabolism. Finally, rigorous *in vivo* testing using atherosclerosis-prone animal models (such as ApoE−/− or LDLR−/− mice) is essential to validate actual plaque reduction, systemic bioavailability, off-target safety, and overall therapeutic efficacy. Ultimately, this research strongly supports the United Nations Sustainable Development Goals specifically SDG 3 (Good Health and Wellbeing) and SDG 9 (Industry, Innovation, and Infrastructure). By providing an innovation-driven, scalable roadmap for the discovery of plant-derived therapeutic agents, this study establishes a vital scientific foundation for developing safer, more affordable, and globally accessible multi-target treatments for cardiovascular disease.

## Data Availability

The datasets presented in this study can be found in online repositories. The names of the repository/repositories and accession number(s) can be found in the article/Supplementary Material.
