# Supplementary material for: Computational validation and network pharmacology reveal the cardioprotective and hypolipidemic potential of Arisaema Jacquemontii Blume via molecular docking, metadynamics, DFT, and MM/PBSA analyses
Source: Front Bioinform. 2026 Jun 12;6:1856040. doi: 10.3389/fbinf.2026.1856040 (PMC13303934; doi:10.3389/fbinf.2026.1856040)
Supplement: Supplementary file 1 [file DataSheet1.pdf]

Computational Validation and Network Pharmacology Reveal the Cardioprotective and Hypolipidemic Potential of *Arisaema Jacquemontii Blume* via Molecular Docking, Metadynamics, DFT, and MM/PBSA Analyses

Manisha Shah[1], Sivakumar Arumugam[1]*

*Correspondence: siva_kumar.a@vit.ac.in

1. Department of Bio-Sciences, School of Bio Sciences and Technology, Vellore Institute of Technology Vellore, Tamil Nadu, 632014, India.

Supplementary Files

Table 1: Different phytochemicals in various parts of *A. Jacquemontii Blume*.

SL No.	Phytochemical Name	SMILES	PubChem ID
1	2-Fluoro-6- (trifluoromethyl) - acetophenone	CC(=O)C1=C(C=CC=C1F)C(F)(F)F	519414
2	Triallylmethylsilane	C[SiH](C)C	70435
3	Propanenitrile, 3- (methylthio)	CSCCC#N	548386
4	Octane, 1-(propylthio)-	CCCCCCCCSCCC	520831
5	2, 5- dimethyl-3-isopropylpyrazine	CC1=CN=C(C(=N1)C(C)C)C	518790
6	Phenol, 2, 5- bis (1,1-dimethylethyl)-	CC(C)(C)C1=CC(=C(C=C1)C(C)(C)C)O	79983
7	Pentadecanoic acid, methyl ester	CCCCCCCCCCCCCCC(=O)O C	23518
8	Ortho-Methoxyacetophenone	CC(=O)C1=CC=CC=C1OC	68481
9	4'- Diethylaminoacetanilide	CCN(CC)C1=CC=C(C=C1)NC (=O)C	21400
10	Nonanoic acid, methyl ester	CCCCCCCCC(=O)OC	15606
11	2,5-Dimethylcyclohexanol	CC1CCC(C(C1)O)C	97959
12	Beta, - methyl xyloside	CC1(C(C(COC1O)O)O)O	129633168
13	Nonanoic Acid	CCCCCCCCC(=O)O	8158
14	3- decanol	CCCCCCCC(CC)O	519158
15	Phenol, 2, 5- bis (1,1-dimethylethyl)-	CC(C)(C)C1=CC(=C(C=C1)C(C)(C)C)O	79983
16	Benzene, 2-methyl-1, 3, 5-trimethyl	CC1=CC(=C(C(=C1)C)SC)C	11084258
17	Nonanedioic acid, dimethyl ester	COC(=O)CCCCCCCC(=O)OC	15612
18	Tridecanoic acid, methyl ester	CCCCCCCCCCCCC(=O)OC	15608
19	n- Hexadecanoic acid	CCCCCCCCCCCCCCCC(=O) O	985
20	Octadecanoic acid, methyl ester	CCCCCCCCCCCCCCCCCC(= O)OC	8201
21	p- Hexyloxy nitro benzene	CCCCCCOC1=CC=C(C=C1)[N+](=O)[O-]	84912

22	Gamma-Sitosterol	CCC(CCC(C)C1CCC2C1(CCC3C2CC=C4C3(CCC(C4)O)C)C)C(C)C	457801

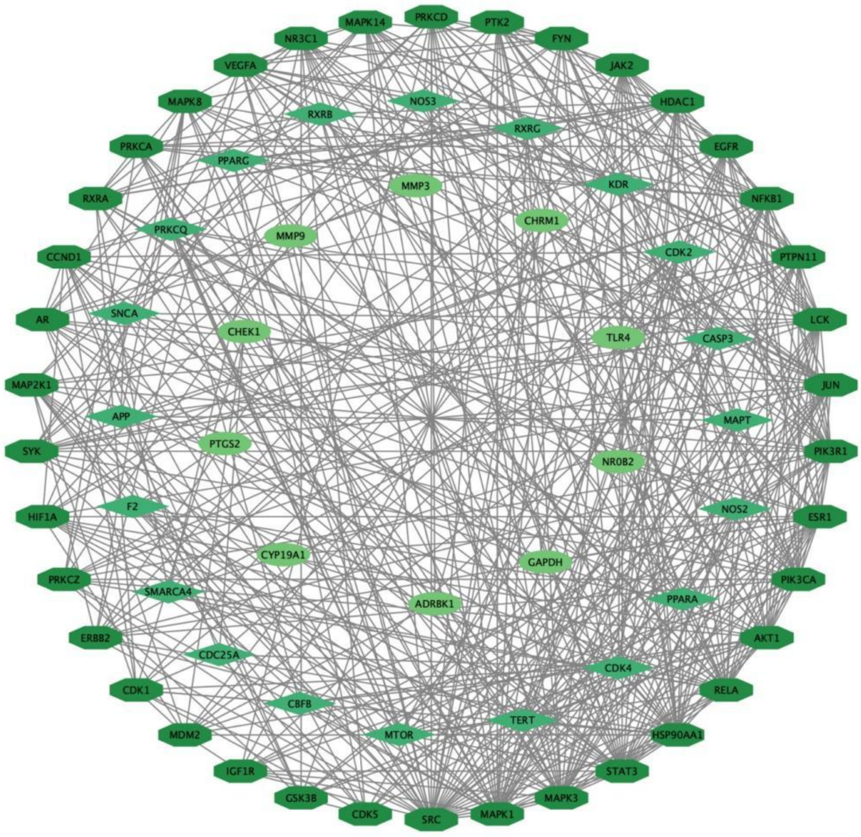

Figure 1: A visual representation of the protein-protein interaction network constructed using the CytoNCA plugin of Cytoscape. Nodes represent proteins, and edges represent interactions between proteins. The darker green color nodes correspond to their degree centrality, indicating the number of interaction.

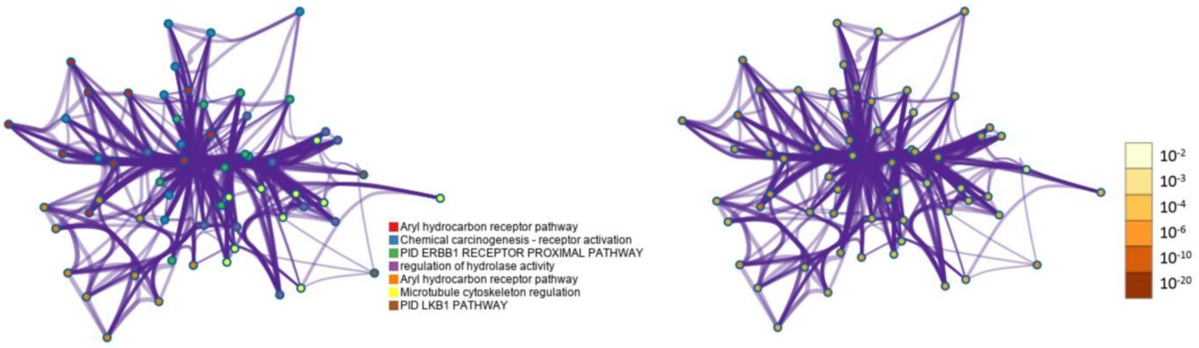

Figure 2: Network of enriched terms. (a) coloured by cluster ID, where nodes that share the same cluster ID are typically close to one another; (b) coloured by p-value, where terms containing more genes tend to have a more significant p-value.

Table 2: Top 10 GO annotations in Metascape

GO	Category	Description	Count	%	Log10(P)	Log10(q)
WP2586	WikiPathways	Aryl hydrocarbon receptor pathway	4	66.67	-10.16	-5.98
hsa05207	KEGG Pathway	Chemical carcinogenesis- receptor activation	5	83.33	-10.02	-5.98
M127	Canonical Pathway	PID ERBB1 RECEPTOR PROXIMAL PATHWAY	3	50	-7.55	-4.19
GO:0051336	GO Biological Processes	Regulation of hydrolase activity	5	83.33	-7.28	-4.05
WP2873	WikiPathways	Aryl hydrocarbon receptor pathway	3	50	-7.21	-4.04
WP2038	WikiPathways	Microtubule cytoskeleton regulation	3	50	-7.18	-4.04
M87	Canonical Pathway	PID LKB1 PATHWAY	3	50	-7.16	-4.04

Table 3: Binding affinity of the active ingredient with TNF-alpha protein

SL No.	Active Ingredient	Canonical SMILE	PubChem ID	Binding affinity
1	2-Fluoro-6-(trifluoromethyl)-acetophenone	CC(=O)C1=C(C=CC=C1F)C(F)(F)F	519414	-4.8
2	2, 5-Dimethyl-3-isopropylpyrazine	CC1=CN=C(C(=N1)C(C)C)C	518790	-4.2
3	Phenol, 2, 5- bis (1,1-dimethylethyl)-	CC(C)(C)C1=CC(=C(C=C1)C(C)(C)C)O	79983	-5.3
4	Pentadecanoic acid, methyl ester	CCCCCCCCCCCCCCC(=O)OC	23518	-4.1
5	Ortho-Methoxyacetophenone	CC(=O)C1=CC=CC=C1OC	68481	-4.6
6	4'- Diethylaminoacetanilide	CCN(CC)C1=CC=C(C=C1)NC(=O)C	21400	-4.7
7	Nonanoic acid, methyl ester	CCCCCCCCC(=O)OC	15606	-3.9
8	2,5-Dimethylcyclohexanol	CC1CCC(C(C1)O)C	97959	-4.2
9	Beta, - methyl xyloside	CC1(C(C(COC1O)O)O)O	129633168	-4.2
10	Nonanoic Acid	CCCCCCCCC(=O)O	8158	-4.2
11	3- decanol	CCCCCCCC(CC)O	519158	-3.9
12	Benzene, 2-methyl-1, 3, 5-trimethyl	CC1=CC(=C(C(=C1)C)SC)C	11084258	-4.4
13	Nonanedioic acid, dimethyl ester	COC(=O)CCCCCCCC(=O)OC	15612	-3.9
14	Tridecanoic acid, methyl ester	CCCCCCCCCCCCC(=O)OC	15608	-4
15	n- Hexadecanoic acid	CCCCCCCCCCCCCCCC(=O)O	985	-4.1
16	Octadecanoic acid, methyl ester	CCCCCCCCCCCCCCCCCC(=O)OC	8201	-4.4
17	p- Hexyloxy nitro benzene	CCCCCCOC1=CC=C(C=C1)[N+](=O)[O-]	84912	-4.9

18	Gamma-Sitosterol	CCC(CCC(C)C1CCC2C1(CCC3C2CC=C4C3(CCC(C4)O)C)C)C(C)C	457801	-7
19	Native ligand	CC1=CC2=C(C=C1C)OC=C(C2=O)CN(C)CCN(C)CC3=CN(C4=CC=CC=C43)C5=CC=CC(=C5)C(F)(F)F	5327044	-5.7

Table 4: Binding affinity of the active ingredient with the ESR1 protein

SL No.	Active Ingredient	Canonical SMILE	PubChem ID	Binding affinity
1	2-Fluoro-6-(trifluoromethyl)-acetophenone	CC(=O)C1=C(C=CC=C1F)C(F)(F)F	519414	-6.5
2	2, 5-Dimethyl-3-isopropylpyrazine	CC1=CN=C(C(=N1)C(C)C)C	518790	-5.2
3	Phenol, 2, 5- bis (1,1-dimethylethyl)-	CC(C)(C)C1=CC(=C(C=C1)C(C)(C)C)O	79983	-6.7
4	Pentadecanoic acid, methyl ester	CCCCCCCCCCCCCCC(=O)OC	23518	-5.4
5	Ortho-Methoxyacetophenone	CC(=O)C1=CC=CC=C1OC	68481	-5.6
6	4'- Diethylaminoacetanilide	CCN(CC)C1=CC=C(C=C1)NC(=O)C	21400	-6.1
7	Nonanoic acid, methyl ester	CCCCCCCCC(=O)OC	15606	-4.9
8	2,5-Dimethylcyclohexanol	CC1CCC(C(C1)O)C	97959	-5.5
9	Beta, - methyl xyloside	CC1(C(C(COC1O)O)O)O	129633168	-5
10	Nonanoic Acid	CCCCCCCCC(=O)O	8158	-4.9
11	3- decanol	CCCCCCCC(CC)O	519158	-5.2
12	Benzene, 2-methyl-1, 3, 5-trimethyl	CC1=CC(=C(C(=C1)C)SC)C	11084258	-5.9
13	Nonanedioic acid, dimethyl ester	COC(=O)CCCCCCCC(=O)OC	15612	-5
14	Tridecanoic acid, methyl ester	CCCCCCCCCCCCC(=O)OC	15608	-5.4
15	n- Hexadecanoic acid	CCCCCCCCCCCCCCCC(=O)O	985	-5.6
16	Octadecanoic acid, methyl ester	CCCCCCCCCCCCCCCCCC(=O)OC	8201	-6.2
17	p- Hexyloxy nitro benzene	CCCCCCOC1=CC=C(C=C1)[N+](=O)[O-]	84912	-5.9
18	Gamma-Sitosterol	CCC(CCC(C)C1CCC2C1(CCC3C2CC=C4C3(CCC(C4)O)C)C)C(C)C	457801	-5.7
19	Native ligand	C[C@H]1CCN(C1)CCOC2=CC=C(C=C2)[C@H]3[C@H](CCC4=C3C=CC(=C4)O)C5=CC=CC=C5	145865316	-6.0

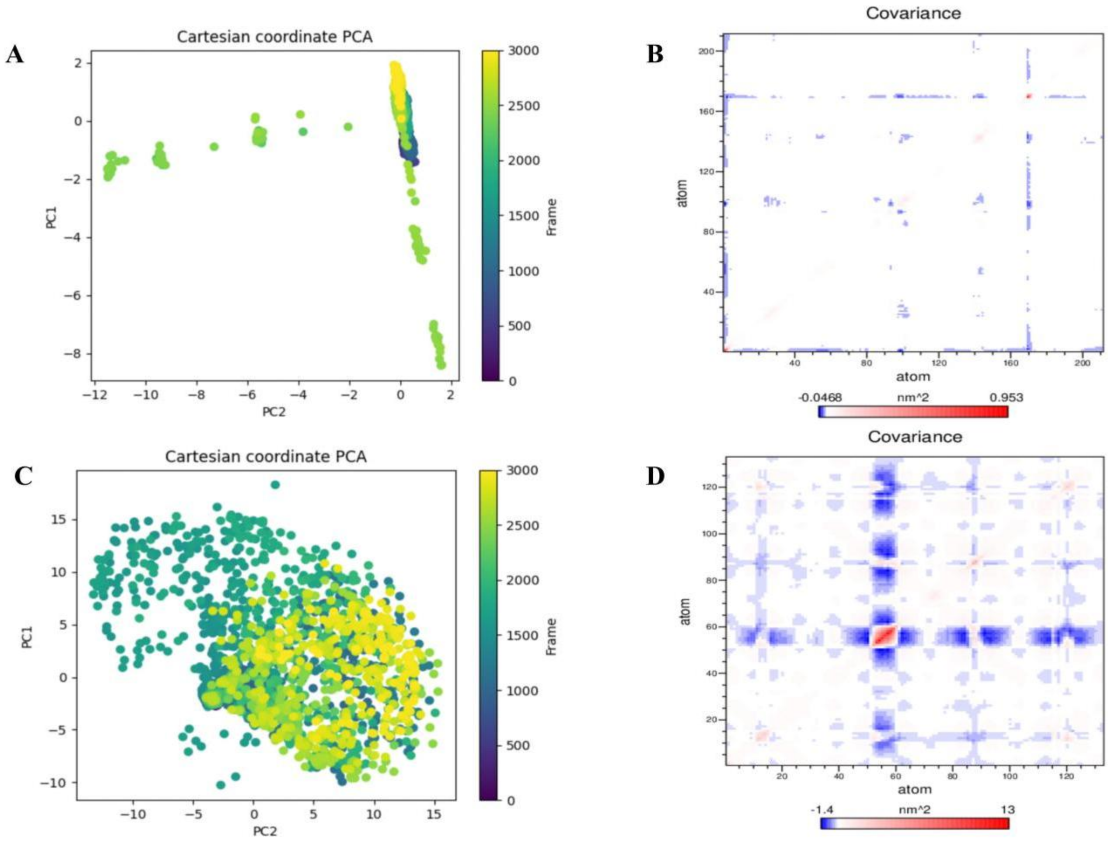

Figure 3: Principal Component Analysis (PCA) and Dynamic Cross-Correlation Matrix (DCCM) plots for ESR1–standard and TNF-alpha–standard complexes. (**A**) PCA plot (left) and (**B**) DCCM map (right) for the ESR1 receptor bound to standard (native ligand), illustrating the dominant motions (PC1 vs. PC2) and residue correlation patterns. (**C**) PCA plot (left) and (**D**) DCCM map (right) for TNF-alpha bound to standard (native ligand), showing principal component distribution and correlated (red) and anti-correlated (blue) atomic motions during the simulation.

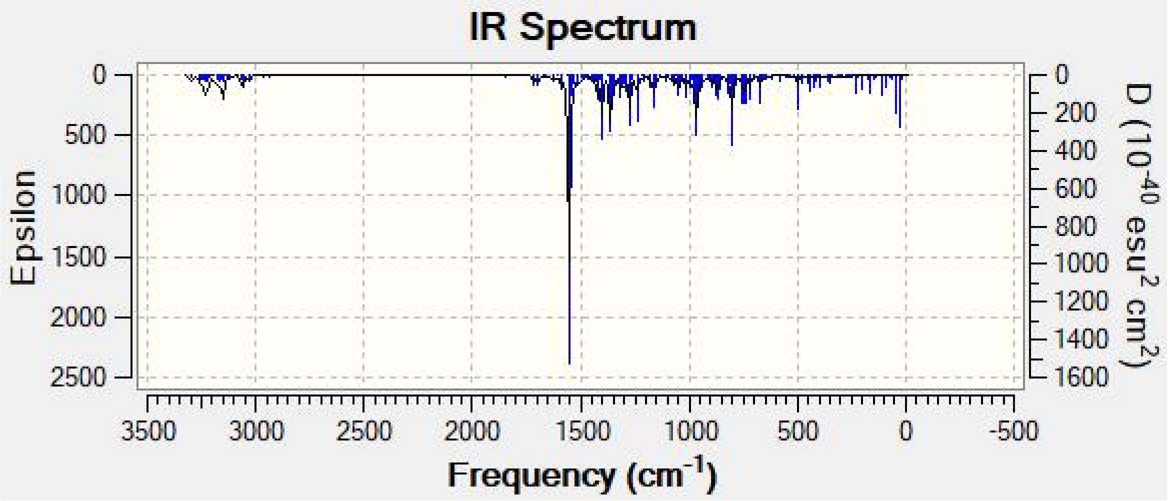

Figure 4: IR spectrum of Gamma-sistosterol at B3LYP/6-31G(d)

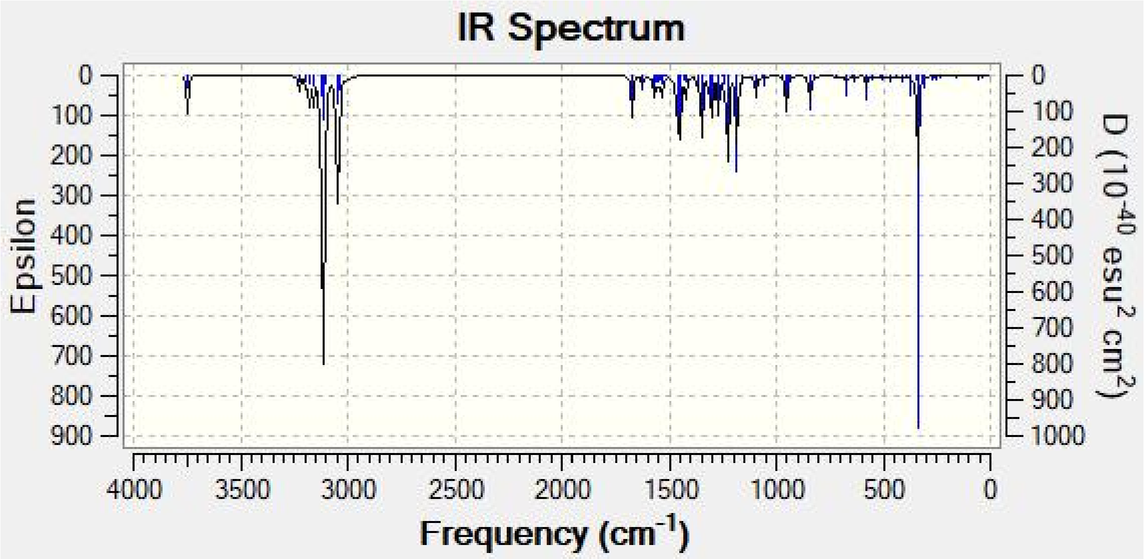

Figure 5: IR spectrum of Phenol 2,5-bis(1,1-dimethylethyl)- at B3LYP/6-31G(d)

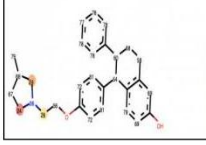

3A4 Ranking	Atom	3A4 Score	Energy	2DSASA	Span2end	Relative Span	Similarity
1	C.4	24.4	32.8	34.9	2	0.9	1.0
2	C.6	24.5	32.8	32.7	2	0.9	0.3
3	C.7	25.8	32.8	24.5	4	0.8	0.4

Native Ligand

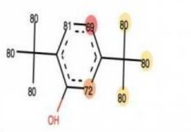

3A4 Ranking	Atom	3A4 Score	Energy	2DSASA	Span2end	Relative Span	Similarity
1	C.10	68.6	74.1	22.0	3	0.6	0.7
2	C.6	71.8	77.2	19.8	3	0.6	0.3
3	C.1	79.5	89.6	53.6	0	1.0	0.3

Phenol, 2, 5- bis (1,1-dimethylethyl)-

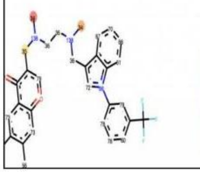

3A4 Ranking	Atom	3A4 Score	Energy	2DSASA	Span2end	Relative Span	Similarity
1	C.16	33.8	41.1	53.3	7	0.6	0.7
2	C.20	34.2	41.1	53.3	8	0.6	0.7
3	C.14	35.1	41.1	20.0	7	0.6	0.4

Native Ligand

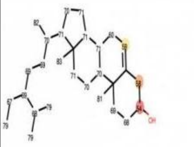

3A4 Ranking	Atom	3A4 Score	Energy	2DSASA	Span2end	Relative Span	Similarity
1	C.23	54.3	62.2	11.1	1	0.9	0.3
2	C.24	58.5	66.4	24.6	2	0.9	0.3
3	C.18	59.0	65.6	19.5	4	0.7	0.3

Gamma-sitosterol

Figure 6: Predicted metabolic reactivity of Phenol, 2,5-bis(1,1-dimethylethyl)- and Gamma-sitosterol, along with their corresponding native ligands (standards), in relation to cytochrome P450 3A4 metabolism, as analyzed using the SMARTCyp 3.0 server.
